# Mechanistic insights into the Bushen Huatan Huoxue Formula and its components in ameliorating obesity-associated polycystic ovary syndrome

**DOI:** 10.1186/s13020-025-01165-3

**Published:** 2025-07-01

**Authors:** Yu-Xin Jin, Hang-Qi Hu, Jia-Cheng Zhang, Yu-Tian Zhu, Hao-Lin Zhang, Xi-Yan Xin, Rui-Wen Fan, Yang Ye, Yin Li, Dong Li

**Affiliations:** 1 State Key Laboratory of Female Fertility Promotion, Department of Traditional Chinese Medicine, Peking University Third Hospital, Beijing, China; 2 Department of Integration of Chinese and Western Medicine, School of Basic Medical Sciences, Peking University, Beijing, China

**Keywords:** Polycystic ovary syndrome, Bushen Huatan Huoxue Formula, Obesity, Metabolic regulation, Mitochondrial function

## Abstract

**Background:**

Bushen Huatan Huoxue Formula (BHHF), a traditional Chinese medicine decoction, demonstrates potential in treating polycystic ovary syndrome (PCOS), a prevalent endocrine disorder in women of reproductive age that is closely associated with obesity and metabolic dysregulation. However, the underlying molecular mechanisms of BHHF’s action remain unclear. This study aimed to evaluate the therapeutic efficacy of BHHF in obesity-associated PCOS and investigate its regulatory mechanisms related to metabolic homeostasis.

**Methods:**

In vivo, three-week-old female Sprague Dawley rats were divided into seven groups: control, dehydroepiandrosterone (DHEA), high-fat diet (HFD), model (HFD + DHEA), low-dose BHHF, high-dose BHHF, and metformin. The PCOS model was induced by DHEA injection. BHHF was administered by gastric gavage for four weeks. Body weight, fat volume, glucose tolerance, and insulin sensitivity were measured. Ovarian histology, hormone analysis, RNA extraction, quantitative real-time PCR, protein extraction, western blotting, and proteomics studies were also conducted. In vitro, 3T3-L1 cells were used to assess lipid accumulation, mitochondrial function, and the effects of BHHF-containing serum.

**Results:**

BHHF restored reproductive cyclicity and polycystic ovarian morphology, reduced testosterone and anti-Müllerian hormone levels, and increased estradiol levels. It also alleviated weight gain, reduced fat volume, improved glucose tolerance, and enhanced insulin sensitivity. Proteomics analysis revealed that BHHF activated the AMPK signaling pathway and promoted white adipose tissue browning. In vitro, BHHF-containing serum suppressed lipid accumulation and enhanced mitochondrial oxygen consumption. The bioactive components of BHHF–Bushen (BS), Huatan (HT), and Huoxue (HX) –exhibited specific functions. BS improved estrous cyclicity and ovarian morphology; HT regulated glucose and lipid metabolism and promoted adipose browning; and HX modulated mitochondrial bioenergetics and redox homeostasis.

**Conclusion:**

BHHF exerts multi-targeted therapeutic effects on obesity-associated PCOS by regulating metabolic-reproductive crosstalk. Its components act synergistically, offering a novel therapeutic strategy for PCOS treatment. Future research should focus on identifying core active compounds and optimizing treatment according to individual PCOS phenotypes.

**Supplementary Information:**

The online version contains supplementary material available at 10.1186/s13020-025-01165-3.

## Introduction

Polycystic ovary syndrome (PCOS), a multifaceted endocrine–metabolic disorder affecting 6–20% of women of reproductive age worldwide [1, 2], presents as the diagnostic triad of hyperandrogenism driven by ovarian theca cell dysfunction [3, 4], ovulatory dysregulation associated with hypothalamic–pituitary–ovarian axis abnormalities [5, 6], and polycystic ovarian morphology. It concurrently predisposes individuals to severe metabolic complications [7]. Beyond its reproductive implications, PCOS is strongly associated with systemic glucolipid metabolic dysregulation [8], including insulin resistance (IR) [9], dysfunctional adipose tissue plasticity [10], mitochondrial bioenergetic insufficiency, [11, 12] and the subsequent development of type 2 diabetes mellitus [13], non-alcoholic fatty liver disease [14, 15], and cardiovascular disease [16]. Central to this metabolic dysregulation is adipose tissue dysfunction, where hypertrophic white adipocytes exhibit blunted lipolysis and impaired thermogenic capacity. These changes exacerbate ectopic lipid deposition and hyperinsulinemia, creating a vicious cycle that perpetuates ovarian hyperandrogenemia via steroidogenic hyperactivation [17–19].

Despite advances in pharmacological treatments–including GLP-1 receptor agonists [20, 21], metformin [22], rosiglitazone, pioglitazone [23], and inositol [24]–current therapies typically address either metabolic or reproductive symptoms, without achieving a comprehensive resolution. Moreover, long-term adherence is limited by adverse effects, ranging from gastrointestinal discomfort to reduced bone mineral density [25]. These limitations underscore the need for multitarget approaches that simultaneously address hypothalamic–pituitary–ovarian axis dysfunction and adipose–liver crosstalk. The integration of Traditional Chinese Medicine (TCM) has emerged as a promising strategy for addressing the heterogeneity of PCOS [26–28]. For example, berberine, a monomer derived from *Coptis chinensis*, improves insulin resisitance via AMPK-dependent GLUT4 translocation [29, 30], while resveratrol, from *Polygonum cuspidatum*, enhances ovarian steroidogenesis by suppressing CYP17A1 overactivity [31]. However, current TCM research often employs reductionist approaches that focus on single compounds (e.g., berberine, resveratrol), overlooking formula–level synergism that is essential for regulating multi-organ crosstalk. This is particularly relevant given the involvement of the hypothalamic–pituitary–ovarian, adipose–liver, and gut–microbiota axes in PCOS pathophysiology. Recent network pharmacology analyses have shown that TCM formulas such as Guizhi Fuling Wan regulated more than 120 PCOS-related targets, influencing pathways such as PI3K/AKT insulin signaling [32, 33], NLRP3 inflammasome activation, and autophagy via the H19/miR-29b-3p axis [34]. Nevertheless, the mechanistic validation of these multitarget effects–particularly in relation to metabolic–reproductive crosstalk–remains insufficient.

Bushen Huatuan Huoxue Formula (BHHF), a TCM empirical prescription for treating PCOS, has demonstrated clinical efficacy [35]. Our previous studies showed that Bushen Huatan Granules improved PCOS-like symptoms induced by dehydroepiandrosterone (DHEA) in rats, potentially through the regulation of mitochondria-dependent apoptosis of granulosa cells [36]. However, the precise mechanisms underlying the action of the BHHF remain unclear. Therefore, this study aimed to explore the different mechanisms of the Bushen (BS), Huatan (HT), and Huoxue (HX) components within BHHF by applying a formula decomposition approach. This investigation may provide insight into the scientific rationale for the synergistic combination of ingredients in BHHF.

## Materials and methods

### Reagents and antibodies

Yin Yang Huo (*Epimedium brevicornum* Maxim.), Nv Zhen Zi (*Ligustrum lucidum* W.T.Aiton), Tu Si Zi (*Cuscuta chinensis* Lam.), Cang Zhu (*Atractylodes lancea* (Thunb.) DC.), Huang Lian (*Coptis chinensis* Franch.), Dan Nan Xing (*Arisaema erubescens*), Xiang Fu (*Cyperus rotundus* L.), Dan Shen (*Salvia miltiorrhiza* Bunge), and Bai Shao (*Paeonia lactiflora* Pall.) were purchased from Beijing Tongrentang Co., Ltd. (Beijing, China). Professor Dong Li confirmed the identity and quality of the herbs. The AMPK inhibitor was obtained from MedChemExpress (New Jersey, USA). Primary antibodies against uncoupling protein 1 (UCP1), carnitine palmitoyltransferase 1α (CPT1α), peroxisome proliferator-activated receptor-γ coactivator 1-α (PGC1α), and β-actin were acquired from Abcam (Cambridge, UK). Primary antibodies against NDUFA6, NDUFA10, NDUFA12, phospho-AMPKα (Thr172), and AMPKα were purchased from Cell Signaling Technology (Danvers, USA). Item numbers and dilution ratios are described in the subsequent methodological sections.

### Preparation of BHHF and compounds identification

BHHF consists of nine herbs: Yin Yang Huo, Nv Zhen Zi, Tu Si Zi, Cang Zhu, Huang Lian, Dan Nan Xing, Xiang Fu, Dan Shen and Bai Shao (Table S1). The herbs were soaked in distilled water and decocted. The resulting decoction was filtered through gauze to remove residue and was stored for immediate use. The clinically recommended dosage for a 70 kg adult is 107 g/day. The equivalent doses for rats, based on body surface area conversion, is 6.3 times higher (9.6 g/kg/day).

A UPLC-QE-Orbitrap-MS/MS (UPLC-MS) system was employed for quality control and compound identification. The chromatographic system used was an ACQUITY UPLC I-Class system, coupled with a Q-Exactive quadrupole-Orbitrap mass spectrometer. BHHF samples were diluted with pure water, and L-2-chlorophenylalanine was used as the internal standard. After centrifugation at 13,000 rpm for 10 min at 4 °C, the supernatant was filtered through a 0.22 μm membrane. Separation was performed using a Waters ACQUITY UPLC HSS T3 column (100 mm × 2.1 mm, 1.8 μm), with 0.1% formic acid in water (mobile phase A) and acetonitrile (mobile phase B). The column temperature was maintained at 30 °C, and the injection volume was 5 μL with a flow rate of 0.3 mL/min. A gradient elution program was applied as follows: 0–5 min, 5% B; 5–10 min, 20% B; 10–14.5 min, 25% B; 14.5–20 min, 30% B; 20–23 min, 50% B; 23–26 min, 80% B; 26–28 min, 90% B; 28–30 min, 95% B; 30–32 min, 100% B. The mass scan range was set to 100–1500 m/z, with a resolution of 17,500 for MS/MS scans. The ion spray voltage was set to 5.5 kV in positive mode and − 4.5 kV in negative mode. The temperature was maintained at 320 °C. Thermo Xcalibur™ software was used for data processing, and Compound Discoverer software was employed for compound identification.

### Animals

Three-week-old female Sprague Dawley (SD) rats (50 ± 5 g) were obtained from the Department of Laboratory Animal Science, Peking University Health Science Center (Beijing, China). All rats were housed in standard plastic rodent cages under regulated conditions (24 °C; 12-h light/dark cycle) with ad libitum access to a standard rat chow and water and food. All animal procedures were approved by the Animal Care and Use Committee of Peking University.

### Experimental group design

After one week of acclimatization, the rats were randomly divided into seven groups:

Control group: normal chow diet (10 kcal% fat, NCD, Research Diets, USA) + sesame oil (vehicle for DHEA) + saline (vehicle for BHHF); DHEA group: NCD + DHEA + saline; High-fat diet group (HFD): high-fat diet (45 kcal% fat, D12451, HFD, Research Diets, USA) + sesame oil + saline; Model group: HFD + DHEA + saline; Low-dose BHHF (BHHF-L) group: HFD + DHEA + 9.6 g/kg BHHF; High-dose BHHF (BHHF-H) group: HFD + DHEA + 19.2 g/kg BHHF; Metformin group: HFD + DHEA + 0.3 g/kg metformin.

BHHF was administered via gastric gavage once daily at the specified doses for four weeks. Rats in the control, DHEA, HFD, and model groups received an equivalent volume of saline. The low dose of BHHF corresponded approximately to the clinical human dose, while the high dose was twice that amount. The PCOS model was established as previously described by subcutaneously injecting female SD rats with DHEA (6 mg/100 g body weight, dissolved in 0.1 mL sesame oil) once daily for 21 consecutive days [37]. Control and HFD group rats received sesame oil alone using the same method. Beginning on day 14 of the intervention, the estrous cycle was assessed daily by vaginal smear. Body weight was measured weekly.

### Preparation of BHHF-containing serum

Twenty male Sprague Dawley (SD) rats with an average weight of 250 g were obtained from the Animal Department of Peking University Health Science Center and were randomly divided into two groups: a control group and a low-dose BHHF treatment group (9.6 g/kg/day), with 10 rats in each group. The control group received a daily oral gavage of an equivalent volume of saline solution. Both groups were treated twice daily by oral gavage, with a 12-h interval between doses, for a continuous period of 14 days. One hour after the final gavage, blood samples were collected from the abdominal aorta under 1% pentobarbital sodium anesthesia. After standing for 1 h, the samples were centrifuged at 3,000 rpm for 10 min. The supernatant was collected and pooled within each group. The pooled serum was heat-inactivated at high temperature for 30 min, filtered through a 0.22 μm microporous membrane, and stored at −80 °C for further use.

### Histological evaluation of ovaries

Ovaries were harvested from rats under anesthesia induced by intramuscular injection of 20% urethane. The tissues were fixed in 4% paraformaldehyde for 48 h and processed into paraffin sections using standard histological procedures. Ten consecutive 5 μm-thick sections were collected from each ovary, with 50 μm intervals between each section. Sections were stained with hematoxylin and eosin, and follicles were classified and counted under a light microscope (BX512DP70; Olympus, Tokyo, Japan).

### Oral glucose tolerance test

At 12 weeks of age, all animals underwent an oral glucose tolerance test (OGTT). Briefly, rats were fasted for 12 h and then administered glucose was given by oral gavage at a dose of 4 g/kg body weight. Blood glucose levels were measured at 0, 15, 30, 60, 90, and 120 min after glucose administration by collecting blood from the tail tip.

### Insulin tolerance test

At 12 weeks of age, all animals underwent an insulin tolerance test (ITT). Rats were fasted for 6 h and then injected intraperitoneally with insulin at a dose of 1 IU/kg body weight. Blood glucose levels were measured at 0, 15, 30, 60, 90, and 120 min following insulin injection by collecting blood from the tail tip. Plasma insulin levels at 0 min were quantified using a commercial enzyme-linked immunosorbent assay (ELISA) kit (Rat Insulin ELISA Kit, Merck-Millipore Co. Ltd., USA), according to the manufacturer’s protocol. The homeostasis model assessment of insulin resistance (HOMA-IR) was calculated using the formula: [fasting glucose (mmol/L) × fasting insulin (mIU/L)]/22.5. The insulin sensitivity index (HOMA-IS) was calculated as the reciprocal of HOMA-IR: 1/HOMA-IR.

### Magnetic resonance imaging (MRI)

Magnetic resonance measurements were performed using a 3.0 T Magnetom Trio MRI scanner (Siemens AG, Munich, Germany). Prior to scanning, animals were sedated in a chamber with 3% isoflurane in medical air, followed by anesthesia induction and maintenance with 1–2% isoflurane via a customized anesthesia mask. Data reconstruction was performed using MATLAB software (The MathWorks, Inc., Natick, USA). Magnitude and phase images were extracted from the complex image data. The magnitude image was used to filter the phase images and suppress statistical phase noise in low-signal regions.

### RNA extraction and quantitative real-time PCR (RT-qPCR)

Total RNA was extracted using commercial TRIzol reagent (Applygen Technologies Inc., Beijing, China) according to the manufacturer’s protocol. Briefly, tissues were homogenized in 1 mL of TRIzol, followed by the addition of 0.2 mL of chloroform. After vigorous shaking for 15 s and incubation on ice for 5 min, the samples were centrifuged at 12,000 × g for 15 min at 4 °C. The aqueous phase was transferred to a new tube and mixed with isopropanol. RNA pellets were obtained by centrifugation and washed with 75% ethanol in RNase-free double-distilled water (ddH2O). The pellets were then dissolved in DEPC-treated water. The yield and purity of each RNA sample were measured using a NanoDrop OneC spectrophotometer (Thermo Scientific, Waltham, MA, USA). To synthesize complementary DNA (cDNA), 2 μg of total RNA was subjected to DNase digestion and reverse transcription using Hifair^®^ II 1 st Strand cDNA Synthesis SuperMix for qPCR, according to the manufacturer’s instructions. PCR amplification was then performed in a reaction mixture containing Hieff^®^ qPCR SYBR^®^ Green Master Mix, forward primer, reverse primer, template cDNA, and sterile ultra-pure water. Primer sequences for each gene are provided in Table S5. The relative mRNA expression levels of target genes were quantified using the 2-∆∆CT method, with β-actin as the internal reference gene. ∆∆CT was calculated as: ∆CT sample—∆CT β-actin.

### Protein extraction and western blot

Tissues were homogenized in radioimmunoprecipitation assay lysis buffer containing a mixture of protease and phosphatase inhibitors on ice for 30 min. The homogenates were then centrifuged at 12,000 × g for 30 min at 4 °C to collect the supernatants. Protein concentrations were determined using a bicinchoninic acid assay. Equal amounts of protein (40 μg per sample) were separated by 12% sodium dodecyl sulfate–polyacrylamide gel electrophoresis and subsequently transferred onto polyvinylidene fluoride membranes. Membranes were blocked with 5% skim milk in Tris-buffered saline containing Tween-20 (TBST) for 1 h at room temperature, then rinsed three times with TBST (5 min each). Membranes were cut according to molecular weight and incubated with primary antibodies (1:1000 dilution) overnight at 4 °C. After primary antibody incubation, membranes were rinsed three times with TBST (5 min each), incubated with the appropriate secondary antibody for 1 h at room temperature, and rinsed again three times with TBST (10 min each). Protein bands were visualized using an enhanced chemiluminescence detection kit (Applygen Technologies, Beijing, China). Densitometric analysis was performed on X-ray films using a bio-image analysis system (Image-Pro Plus 6.0; Media Cybernetics, Bethesda, MD, USA), and band intensities were quantified using ImageJ software (National Institutes of Health, Bethesda, MD, USA).

### Enzyme-linked immunosorbent assay

Double-antibody sandwich ELISAs (Andygene, Richardson, USA) were conducted to quantify serum levels of insulin, anti-Müllerian hormone (AMH), testosterone, estradiol, follicle-stimulating hormone (FSH), luteinizing hormone (LH), adiponectin, apelin, and omentin. Briefly, serum samples were added to monoclonal antibody-coated wells to form immune complexes. After washing the plates, horseradish peroxidase-labeled avidin was added to bind biotin. Subsequently, substrate and stop solutions were added in accordance with the manufacturer’s instructions. Optical density was measured at 450 nm using a microplate reader (MULTISKAN MK3, Thermo, San Jose, CA, USA).

### Mitochondrial electron transport chain (ETC) complex activity assays

Adipose tissue total protein was adjusted to a final concentration of 5.5 mg/mL. Detergent was added to the sample at a ratio of 1:10 (v/v), mixed thoroughly, and incubated on ice for 30 min. The supernatant was collected following centrifugation at 12,000 × g for 20 min. ETC complex activities were assessed using Elabscience assay kits, following the manufacturer’s protocols with critical modifications: Complex I (NADH dehydrogenase, E-BC-K149-M): Reaction mixture contained 25 μg of mitochondrial protein, 2 mM NADH, and 100 μM ubiquinone. Enzymatic activity was monitored at 340 nm (ε = 6.22 mM-1·cm-1) for 5 min, with 10 μM rotenone used as the inhibitor control; Complex II (Succinate dehydrogenase, E-BC-K150-M): 50 μg of protein was incubated with 20 mM succinate and 50 μM DCPIP. Reduction was measured at 600 nm (ε = 21 mM-1·cm-1), with 10 mM malonate as the inhibitor; Complex III (Ubiquinol–cytochrome c reductase, E-BC-K151-M): Activity was determined by cytochrome c reduction at 550 nm (ε = 18.5 mM-1·cm-1) using 50 μM decylubiquinol and 20 μM cytochrome c; Complex IV (Cytochrome c oxidase, E-BC-K152-M): Oxidation of 50 μM reduced cytochrome c was monitored at 550 nm (ε = 18.5 mM-1·cm-1), with 1 mM potassium cyanide used as the inhibitor; Complex V (ATP synthase, E-BC-K153-M): ATP hydrolysis was measured at 340 nm using 2 mM ATP, with 10 μM oligomycin for baseline correction.

### ELISA for energy metabolism status

Total protein from adipose tissue was extracted using a commercial protein extraction kit (Applygen Technologies, Beijing, China). ATP levels in adipose tissue were quantified using ELISA kits (Andihuatai Technology Co. Ltd., Beijing, China) and measured with a microplate reader (MULTISKAN MK3, Thermo, San Jose, CA, USA) according to the manufacturer’s instructions, as described previously.

### NAD⁺/NADPH quantification and redox ratio analysis

NADH-related metabolites were quantified using Elabscience kits (E-BC-K804-M) following the manufacturer's protocol: Total NAD (NAD +  + NADH): 50 μL of supernatant was incubated with 100 μL of NADH developer (containing alcohol dehydrogenase and diaphorase). The reaction was initiated by adding 10 mM ethanol substrate and monitored at 450 nm for 30 min; NAD^+^-specific quantification: Samples were pre-treated with 20 U/mL NADH oxidase at 37 °C for 1 h to eliminate NADH; Redox ratio calculation: NAD^+^/Total NAD ratio was calculated using the formula: NAD^+^/(NAD^+^  + NADH) × 100%, where [NADH] = [Total NAD] – [NAD^+^].

### Cell culture

3T3-L1 cells, a mouse embryonic fibroblast cell line, were obtained from the American Type Culture Collection (ATCC, Rockville, MD, USA). Cells were cultured in Dulbecco’s Modified Eagle’s Medium (DMEM; Invitrogen, Grand Island, NY, USA) supplemented with 10% fetal bovine serum (FBS; Invitrogen), 100 IU/mL penicillin, and 100 μg/mL streptomycin (Life Technologies, Milan, Italy). Cells were maintained in a humidified incubator at 37 °C with 95% air and 5% CO_2_. When cells reached 80–90% confluence, the medium was replaced with DMEM, and treatments were applied for 48 h as follows: Vehicle group: dimethyl sulfoxide (DMSO) only; Control serum group: DMSO + 10% control serum; BHHF serum group: DMSO + 10% BHHF-containing serum; Compound C group: pre-treatment with 10 μM compound C (an AMPK inhibitor) for 2 h, followed by serum treatment for 48 h.

### Adipogenic differentiation of 3T3-L1

Induction Medium I: DMEM supplemented with 10% fetal bovine serum (FBS; Gibco, 26,140,079), 1 μM dexamethasone (Sigma, D4902), 0.5 mM 3-isobutyl-1-methylxanthine (IBMX; Sigma, I5879), and 1 μg/mL insulin (Sigma, I0516). Days 3–6: Cells were switched to Induction Medium II, consisting of DMEM + 10% FBS with 1 μg/mL insulin only. Day 7 onward: Cells were maintained in Adipocyte Maintenance Medium (DMEM + 10% FBS), refreshed every 72 h. Hormone Stock Solutions: Dexamethasone: 10 mM in DMSO (aliquots stored at − 80 °C; avoid more than three freeze–thaw cycles); IBMX: 500 mM in 0.1 N NaOH (stored at − 20 °C, protected from light); Insulin: 1 mg/mL in 5 mM HCl (filter-sterilized and stored at 4 °C).

### Cell viability assay

3T3-L1 cells were seeded in 96-well plates at a density of 5 × 10^4^ cells/well and incubated for 24 h in DMEM supplemented with 10% FBS at 37 °C in a humidified 5% CO_2_ incubator. Cells were then serum-starved for 12 h. After washing twice with phosphate-buffered saline (PBS), cells were treated with control serum or BHHF-containing serum (5–25%) for 24 h. Cell viability was assessed colorimetrically using the Cell Counting Kit-8 (CCK-8) assay, following the manufacturer’s instructions (DOJINDO, Kumamoto, Japan).

### Oil red O staining

Cells were rinsed three times with PBS and fixed with 4% paraformaldehyde for 15 min at room temperature. After one wash with 60% isopropanol, cells were stained for 1 h at room temperature with filtered Oil Red O solution prepared in 60% isopropanol. Cells were then washed once with 60% isopropanol and once with distilled water. Counterstaining was performed with hematoxylin for 30 s, followed by a final PBS wash for 5 min. Red-stained adipocytes were observed under a light microscope.

### Mitochondrial staining with MitoTracker probes

Cells were cultured on sterile glass-bottom dishes to 70–80% confluence under standard conditions (37 °C, 5% CO_2_). Prior to staining, the medium was replaced with pre-warmed (37 °C) serum-free medium to minimize nonspecific dye aggregation. MitoTracker^™^ Red CMXRos (Thermo Fisher, M7512) was prepared at a final concentration of 100 nM in serum-free medium and protected from light. Cells were incubated with the dye solution for 30 min at 37 °C in a humidified CO_2_ incubator. Following incubation, the dye-containing medium was removed, and cells were gently washed three times with PBS (pH 7.4, 37 °C) to eliminate residual probe. For live-cell imaging, cells were maintained in DMEM (Gibco, A1896701) supplemented with 10% FBS. Images were captured within 2 h post-staining using a confocal microscope (e.g., Zeiss LSM 880) equipped with appropriate filters (MitoTracker Red: excitation/emission = 579/599 nm). Live imaging was performed at 37 °C using a stage-top incubator.

### Proteomics studies

Nine adipose tissue samples from rats (control group, model group, and BHHF group) were collected and stored at − 80 °C until further processing. Protease inhibitor and lysis buffer were added according to the tissue weight. Samples were sonicated five times (5 s/cycle with 10-s intervals between cycles) and centrifuged at 12,000 × g for 15 min at 4 °C. The resulting supernatants were collected in 1.5 mL microcentrifuge tubes. Protein concentrations were measured using the Bradford Protein Assay, following the manufacturer’s protocol. A total of 200 μg of protein from each sample was sent to the Medical and Health Analytical Center of Peking University Health Science Center (Beijing, China) for proteomic analysis. Mass spectrometry data were processed and searched using PEAKS Online software. Proteins with a fold change ≥ 1.5, supported by at least two unique peptides and a p-value < 0.05, were considered differentially expressed proteins (DEPs). DEPs were further analyzed using the Kyoto Encyclopedia of Genes and Genomes (KEGG) pathway enrichment analysis.

### Mitochondrial respirometry

Mitochondrial oxygen consumption was measured polarographically at 37 °C in MiR05 buffer (110 mmol/L D-sucrose, 60 mmol/L lactobionic acid, 20 mmol/L taurine, 20 mmol/L HEPES free acid, 10 mmol/L KH2PO4, 3 mmol/L MgCl2, 0.5 mmol/L EGTA free acid, and 1 g/L fatty acid-free bovine serum albumin [BSA], pH 7.1) using a two-channel high-resolution Oxygraph-2 k instrument (Oroboros Instruments, Innsbruck, Austria). Samples, substrates, inhibitors, and uncoupler were sequentially added in the following order: 1) living cells; 2) 5 μg/mL digitonin; 3) 2 mmol/L malate; 4) 2.5 mmol/L ADP; 5) 10 μmol/L cytochrome c; 6) 10 mmol/L glutamate; 7) 10 mmol/L succinate; 8) carbonyl cyanide 4-(trifluoromethoxy) phenylhydrazone (FCCP) titration to induce maximal respiration; 9) 0.5 μmol/L rotenone; 10) 2.5 μmol/L antimycin A. Baseline oxygen (O2) consumption rates were recorded after the addition of each component. The following respiratory states were assessed: Routine respiration (state ROUTINE): basal O2 consumption in intact cells; OXPHOS respiration (state OXPHOS): ADP-stimulated maximal O2 consumption; Electron transport system capacity (state ET): uncoupled O2 consumption; Residual oxygen consumption (state ROX): nonmitochondrial O2 consumption; Data acquisition and analysis were performed using DatLab software version 4.3.1.15 (Oroboros Instruments, Innsbruck, Austria).

### Statistical analysis

All data are expressed as mean ± standard error of the mean (SEM). Statistical analyses were performed using GraphPad Prism software. Differences between groups were evaluated using one-way analysis of variance (ANOVA), followed by the Bonferroni post hoc test. A p-value of < 0.05 was considered statistically significant.

## Results

### BHHF restored reproductive cyclicity and polycystic ovarian morphology

**Fig. 1:**
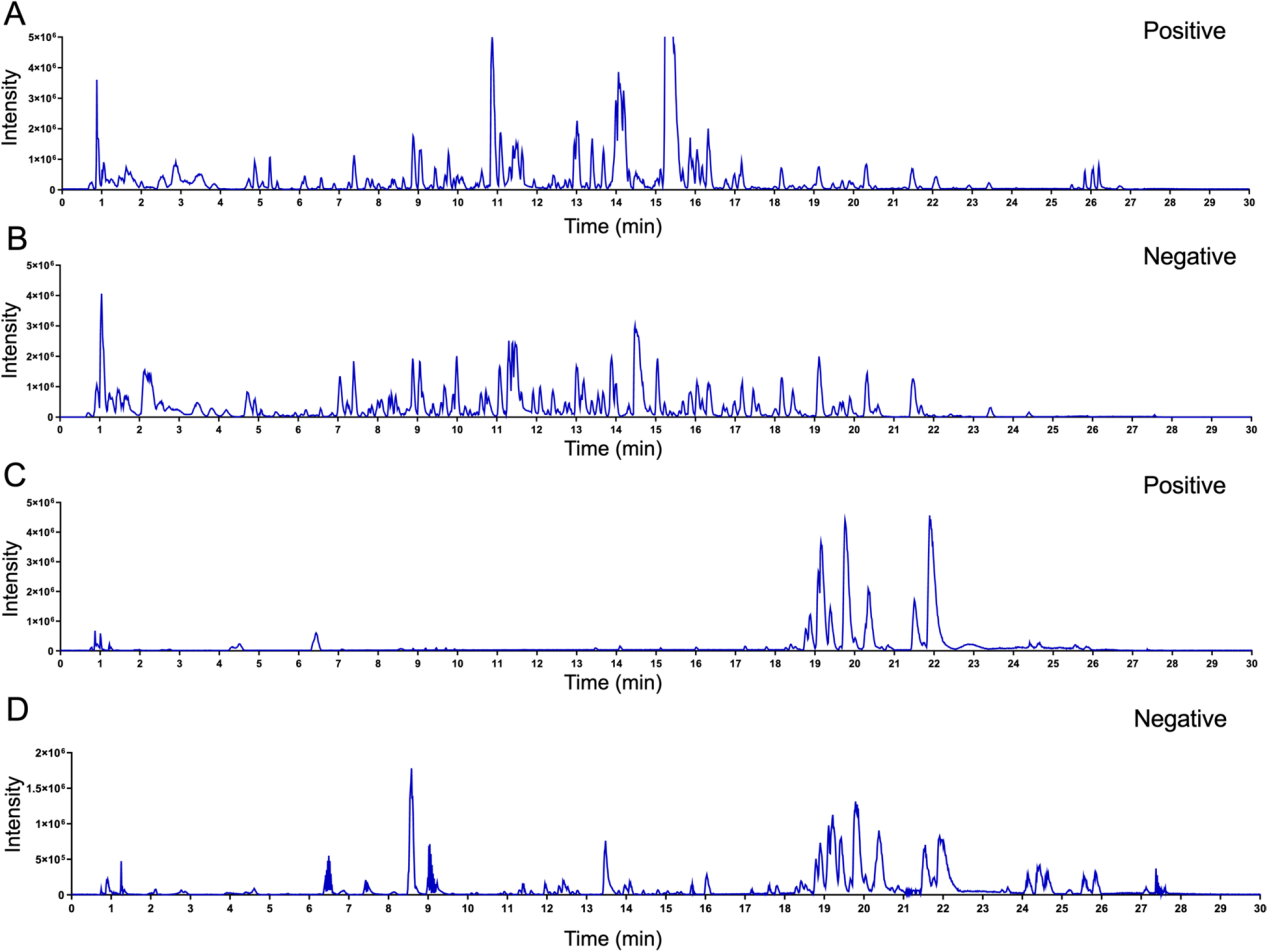
Base peak chromatograms (BPCs) of BHHF and BHHF-containing serum detected by UPLC-MS. **A**: BPC in positive ion mode (BHHF). **B**: BPC in negative ion mode (BHHF). **C**: BPC in positive ion mode (BHHF-containing serum). **D**: BPC in negative ion mode (BHHF-containing serum)

**Fig. 2:**
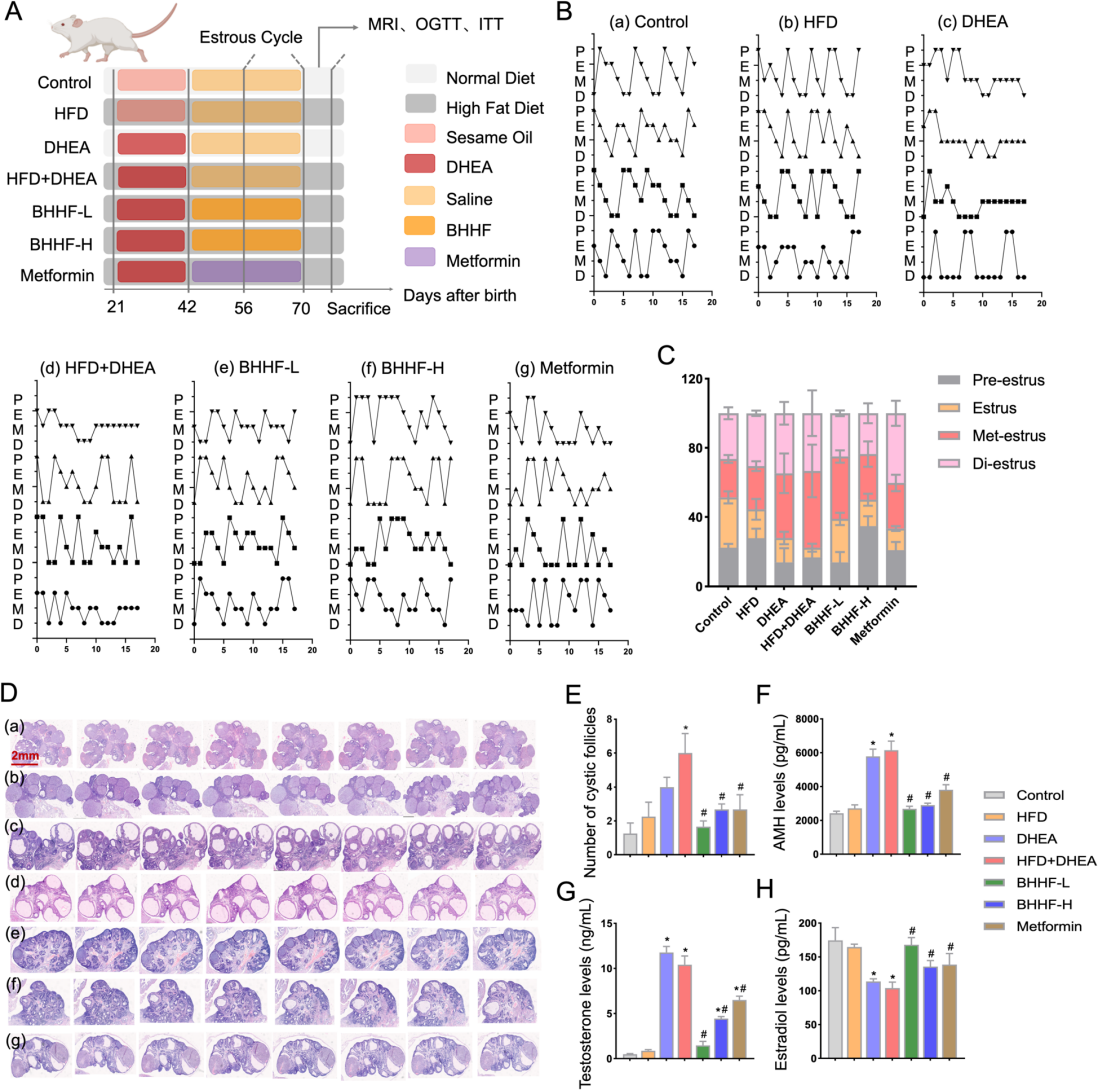
Reproductive cycle analysis, ovarian histomorphology, and endocrine profiling.** A**: Schematic of the PCOS with obesity rat model establishment, experimental grouping, and intervention timeline. **B**: Representative vaginal cytology images depicting estrous cycle phases: proestrus, estrus, metestrus, and diestrus.** C**: Quantitative assessment of estrous cycle regularity. **D**: H&E-stained ovarian sections (scale bar = 2 mm) showing follicular morphology: (**a**) Control, (**b**) HFD, (**c**) DHEA, (**d**) HFD + DHEA, (**e**) BHHF-L, (**f**) BHHF-H, and (**g**) Metformin. **E**: Cystic follicle count per ovarian section. **F**: Serum anti-Müllerian hormone (AMH) levels. **G**: Serum testosterone levels. **H**: Serum estradiol levels. All data are presented as mean ± SEM (n = 4 per group). Statistical significance was determined by one-way ANOVA with Tukey’s post hoc test. (^*^P < 0.05 vs. control; ^#^*P* < 0.05 vs. HFD + DHEA)

UPLC-MS analysis identified multiple bioactive constituents in both the BHHF solution and serum. Cross-validation confirmed the presence of these compounds in both the BHHF formulation and serum (Fig. 1A–D; Tables S1–S3). The experimental timeline and pharmacological intervention strategy are shown in Fig. 2A. Administration of BHHF significantly ameliorated PCOS-associated estrous cycle irregularities. Rats treated with BHHF-L exhibited prolonged estrus duration and reduced frequency of the diestrus phase (Figs. 2B–C). Histopathological analysis revealed significant improvement in polycystic ovarian morphology (PCOM) in both the BHHF- and metformin-treated groups, characterized by a reduction in cystic follicles (Fig. 2E). Systemic hormonal reprogramming indicated that BHHF reversed PCOS-associated endocrine disturbances (Figs. 2F–H): serum testosterone and AMH levels decreased, while estradiol levels increased. Notably, BHHF-L demonstrated comparable efficacy to metformin in follicular normalization (*P* = 0.3804 between groups) and exhibited superior androgen suppression (P < 0.0001 vs. metformin). However, quantitative analysis showed that BHHF treatment did not significantly alter circulating FSH or LH levels across experimental groups (Figures S1D–F).

### BHHF ameliorated systemic glucose and lipid metabolic dysfunction in obese PCOS rats

**Fig. 3:**
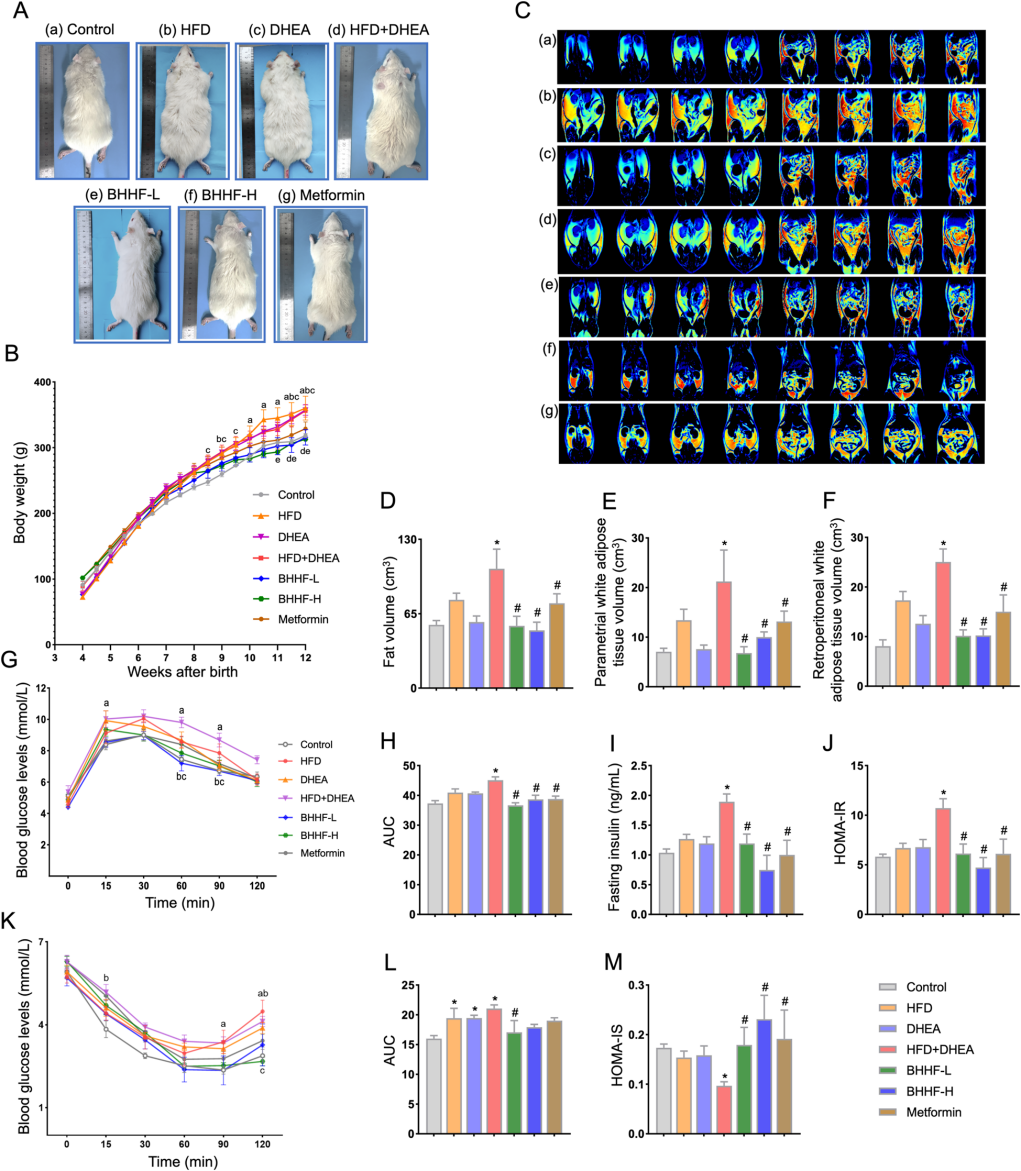
Systemic glucose and lipid metabolic profiles.** A**: Gross morphology of rats. **B**: Longitudinal monitoring of body weight. (a *P* < 0.05, Control vs HFD; b *P* < 0.05, Control vs DHEA; c *P* < 0.05, Control vs HFD + DHEA; d *P* < 0.05, HFD + DHEA vs BHHF-L; e *P* < 0.05, HFD + DHEA vs BHHF-H). **C**: Representative MRI cross-sectional images. (Left four panels: retroperitoneal adipose tissue; right four panels: parametrial adipose tissue.) (a) Control; (b) HFD; (c) DHEA; (d) HFD + DHEA; (e) BHHF-L; (f) BHHF-H; (g) Metformin. **D**: Total fat volume. **E**: Parametrial fat volume. **F**: Retroperitoneal fat volume. **G**: Oral glucose tolerance test (OGTT) curves (0–120 min). (a *P* < 0.05, Control vs HFD + DHEA; b *P* < 0.05, HFD + DHEA vs BHHF-L; c *P* < 0.05, HFD + DHEA vs BHHF-H). **H**: Area under the curve (AUC) of OGTT. **I**: Serum insulin levels after a 12-h overnight fast. **J**: HOMA-IR calculation. **K**: Insulin tolerance test (ITT) curves (0–120 min). (a *P* < 0.05, Control vs HFD; b *P* < 0.05, Control vs HFD + DHEA; c *P* < 0.05, HFD + DHEA vs BHHF-H).** L**: AUC of ITT. **M**: HOMA-IS calculation. All data are presented as mean ± SEM (n = 4 per group). Statistical significance was determined by one-way ANOVA followed by Tukey’s post hoc test (^*^P < 0.05 vs control; ^#^*P* < 0.05 vs HFD + DHEA)

At the end of the 12-week experiment, rats in the model group exhibited significantly increased body weight (Figs. 3A–B) and total fat volume (Fig. 3C) compared with the control group. These increases were attenuated by BHHF treatment, particularly at the low dose. BHHF-L suppressed weight gain by 14.2% relative to the model group (Δ228.7 ± 29.06 g vs. Δ266.7 ± 18.96 g at week 12, *P* = 0.0473), showing comparable efficacy to metformin (Δ227.2 ± 21.62 g, *P* = 0.9378 vs. BHHF-L). To evaluate the effects of BHHF on adipose distribution, parametrial and retroperitoneal white adipose tissue volumes were measured and quantified using MATLAB analysis (Figs. 3D–F). MRI-based quantification demonstrated a systemic reduction in adiposity: total fat volume decreased by 47.8% in the BHHF-L group compared to the model group (*P* = 0.0005); parametrial fat volume was 6.809 ± 2.564 cm^3^ (BHHF-L) versus 21.23 ± 12.66 cm^3^ (model, *P* = 0.001), and retroperitoneal fat volume was 10.18 ± 2.368 cm^3^ (BHHF-L) versus 25.04 ± 5.256 cm^3^ (model, *P* < 0.0001).

To further investigate the role of BHHF in glucose metabolism, OGTTs were conducted. The model group exhibited significantly impaired glucose tolerance and insulin sensitivity. Following glucose loading, blood glucose levels in the model group increased at 15, 30, 60, 90, and 120 min compared with the control group, and the area under the curve (AUC) was significantly higher (Figs. 3G–H). Treatment with BHHF and metformin significantly reduced blood glucose concentrations at 0, 15, 60, and 120 min relative to the model group, and the AUC was significantly decreased (Figs. 3G–H). In addition, ITTs indicated that BHHF-L improved insulin sensitivity, as reflected by reduced AUC values (Figs. 3K–L). As shown in Figs. 3J and 3M, HOMA-IR values decreased from 10.72 ± 2.291 (model) to 6.133 ± 1.926 (BHHF-L, *P* = 0.0004), while HOMA-IS increased from 0.097 ± 0.020 (model) to 0.1793 ± 0.070 (BHHF-L,* P* = 0.0173).

### Network pharmacology analysis of BHHF solution and BHHF serum compounds

**Fig. 4:**
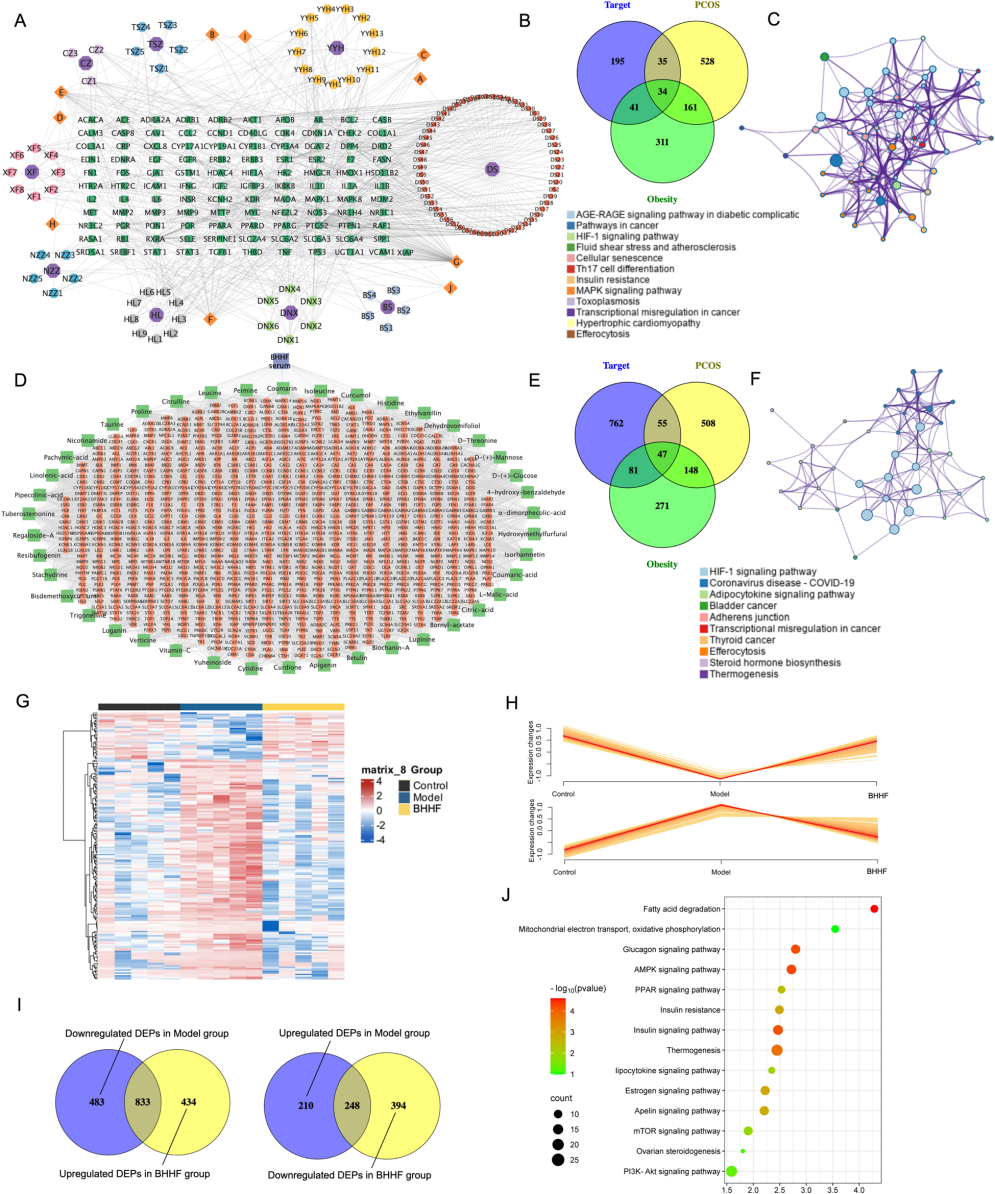
Ingredient analysis, network pharmacology prediction of BHHF solution and serum targets, and proteomic identification of differentially expressed proteins (DEPs) in adipose tissue.** A**: Compound–target network of active components in BHHF. Purple nodes represent the nine ingredients of BHHF; orange nodes represent overlapping chemical components; green nodes represent predicted compound targets. **B**: Venn diagram showing overlap between BHHF target genes and genes associated with PCOS and obesity. **C**: Enriched ontology clusters, colored by cluster ID. **D**: Compound–target network of BHHF serum components. Green nodes represent serum components; orange nodes represent predicted targets. **E**: Venn diagram showing overlap between BHHF serum targets and PCOS/obesity-associated genes. **F**: Enriched ontology clusters, colored by cluster ID. **G**: Heatmap of DEPs in the control, model, and BHHF groups. The color bar indicates the fold change from upregulation to downregulation. **H**: Clustering analysis of DEPs. **I**: Venn diagrams showing overlap between downregulated DEPs in the model group and upregulated DEPs in the BHHF group (n = 833), and vice versa for upregulated DEPs in the model group and downregulated DEPs in the BHHF group (n = 248). **J**: KEGG pathway enrichment analysis for DEPs. Y-axis indicates KEGG pathway names; X-axis indicates the number of proteins involved in each pathway. All data are expressed as mean ± SEM (n = 3 per group). Statistical significance was determined by one-way ANOVA followed by Tukey’s post hoc test (^*^P < 0.05 vs control; ^#^*P* < 0.05 vs HFD + DHEA)

As shown in Figs. 4A–C, potential target genes of the identified compounds were extracted from the Traditional Chinese Medicine Systems Pharmacology database, yielding 305 target genes. A compound–target gene interaction network was constructed for BHHF, highlighting key bioactive compounds such as quercetin, kaempferol, and luteolin, along with core target genes including AKT1, ESR1, and STAT. Comparison of these target genes with disease-associated genes—758 genes related to PCOS and 547 genes associated with obesity from the GeneCards database—identified 34 potential key target genes modulated by BHHF in the treatment of PCOS with obesity (Fig. 4B). Enrichment analysis of biological pathways revealed that AGE–RAGE signaling and insulin resistance were among the key pathways potentially contributing to the reproductive protective effects of BHHF (Fig. 4C). In parallel, the components detected in BHHF serum were subjected to network pharmacology analysis. Their potential protein targets were predicted using the SwissTargetPrediction database, and a corresponding compound–target interaction network was constructed (Fig. 4D). Cross-referencing these targets with PCOS- and obesity-related genes identified 47 key target genes modulated by BHHF serum (Fig. 4E). Subsequent enrichment analysis of the overlapping gene set was performed (Fig. 4F), providing insights into the mechanistic pathways through which BHHF may exert its effects.

### Proteomics analysis identifies the DEPs via label free quantitation (LFQ)

Label-free quantitation (LFQ) proteomics was conducted to assess changes in protein expression among the control, model, and BHHF-L groups. A heatmap was generated based on protein expression profiles across the groups (Fig. 4G). In total, 35,828 valid proteins were identified, each with at least one unique peptide and a false discovery rate below 1%.

Proteins exhibiting a fold change ≥ 1.5 and a p-value < 0.05 were considered DEPs. Between the model and control groups, 1,774 DEPs were identified; between the BHHF-L and model groups, 1,909 DEPs were found. Specifically, 458 proteins were upregulated and 1,316 downregulated in the model group relative to control. Conversely, 1,267 proteins were upregulated and 642 downregulated in the BHHF-L group compared to the model group. Overlap analysis revealed 833 proteins that were downregulated in the model group but upregulated following BHHF-L treatment. Similarly, 248 proteins were upregulated in the model group and subsequently downregulated in the BHHF-L group (Fig. 4I). Cluster enrichment and KEGG pathway analyses were performed on 1,081 overlapping DEPs among the three groups (Fig. 4H–I). The enriched pathways included mitochondrial electron transport, oxidative phosphorylation, AMPK signaling, PPAR signaling, thermogenesis, adipocytokine signaling, and apelin signaling. Of note, regulation of the adipocytokine signaling pathway appeared to underlie BHHF’s beneficial effects on glucolipid metabolism. Therefore, we further examined circulating levels of adiponectin, apelin, and omentin via ELISA. As shown in Figures S1A–B, BHHF-L treatment significantly increased serum apelin and omentin levels compared with the model group.

### Proteomic remodeling of thermogenic pathway and mitochondrial functional validation

**Fig. 5:**
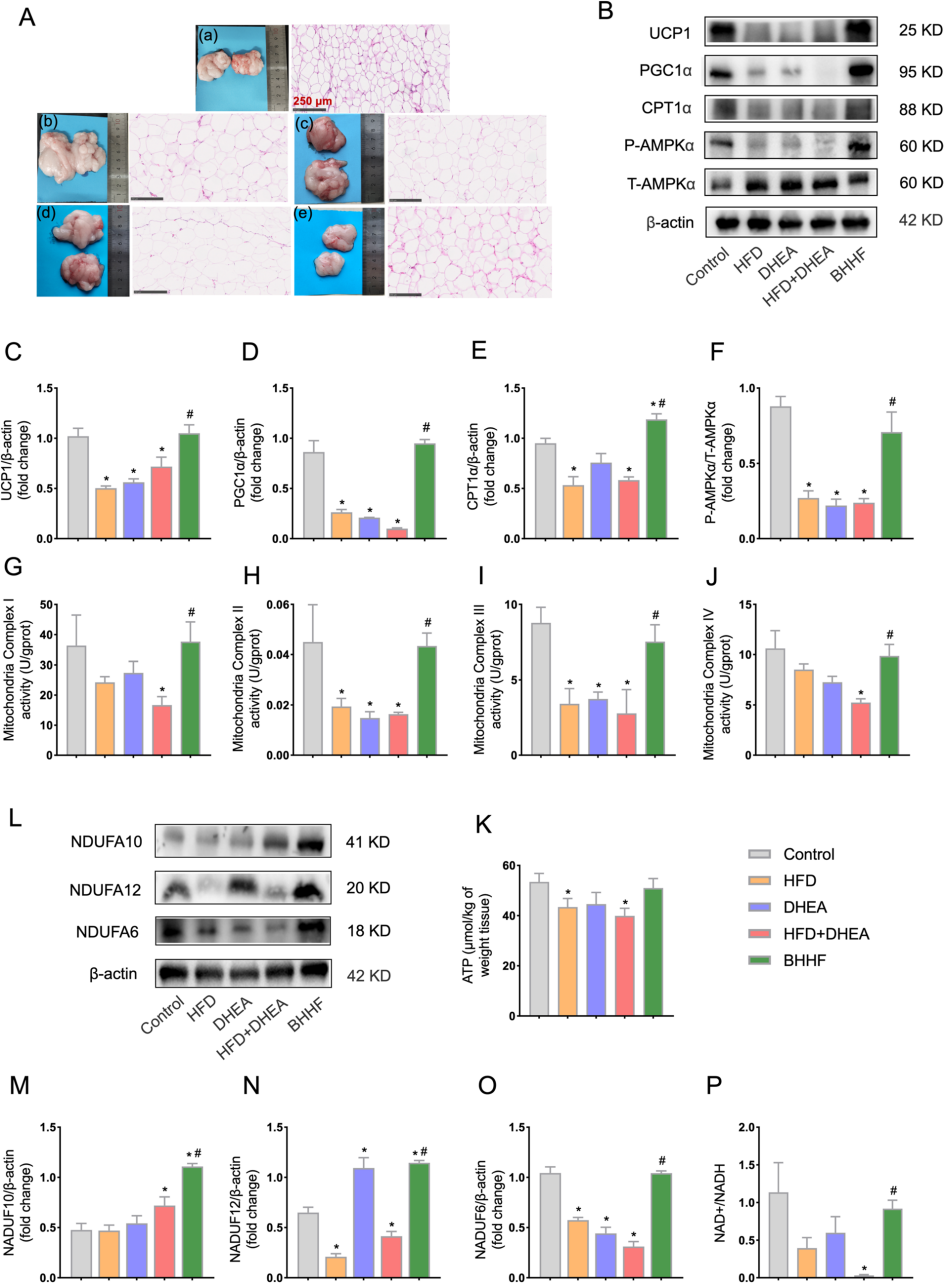
Mitochondrial redox imbalance and thermogenic protein expression in adipose tissue. **A**: Gross morphology of adipose depots (left) and hematoxylin and eosin (H&E) staining (right; scale bar: 250 μm). **B**: Western blot analysis of UCP1, PGC1α, CPT1α, total AMPKα (T-AMPKα), and phosphorylated AMPKα (P-AMPKα). **C–F**: Quantification of protein expression ratios: UCP1/β-actin, PGC1α/β-actin, CPT1α/β-actin, and P-AMPKα/T-AMPKα. **G–J**: Activities of mitochondrial electron transport chain (ETC) complexes in adipose tissue: Complex I (NADH dehydrogenase), Complex II (succinate dehydrogenase), Complex III (ubiquinol–cytochrome c reductase), and Complex IV (cytochrome c oxidase). **K**: Adipose tissue ATP content.** L**: Western blot analysis of Complex I subunits NDUFA10, NDUFA12, and NDUFA6. **M**–**O**: Quantification of NDUFA10/β-actin, NDUFA12/β-actin, and NDUFA6/β-actin expression ratios. **P**: NAD +/NADH redox ratio in adipose tissue, reflecting redox status. All data are expressed as mean ± SEM (n = 3 per group). Statistical significance was determined by one-way ANOVA with Tukey’s post hoc test (^*^P < 0.05 vs control; ^#^*P* < 0.05 vs HFD + DHEA)

KEGG enrichment analysis of adipose tissue proteomics revealed significant activation of the thermogenesis pathway in BHHF-treated groups, with upregulation of UCP1, CPT1α, and AMPKα compared to the model group. Western blot validation supported these findings: UCP1: 1.050 ± 0.169 (BHHF) vs 0.718 ± 0.190 (model, *P* = 0.0041); PGC1α: 0.950 ± 0.077 (BHHF) vs 0.099 ± 0.015 (model, *P* < 0.0001); CPT1α: 1.188 ± 0.110 (BHHF) vs 0.584 ± 0.061 (model, *P* < 0.0001); and P-AMPKα (Thr172)/T-AMPKα: 0.708 ± 0.267 (BHHF) vs 0.239 ± 0.054 (model, *P* = 0.0004) (Figs. 5B–F). Accordingly, hematoxylin–eosin staining of parametrial adipose tissue revealed reduced adipocyte diameter, indicating a pronounced browning phenotype (Fig. 5A). In line with the proteomic predictions, BHHF restored the activity of ETC Complexes I, II, III, and IV (Figs. 5G–J). Despite this enhancement in ETC flux, ATP levels remained unchanged (Fig. 5K), suggesting that the increased mitochondrial activity was directed toward non-shivering thermogenesis rather than ATP synthesis. KEGG pathway analysis further indicated significant upregulation of oxidative phosphorylation, particularly involving Complex I subunits. BHHF treatment significantly increased expression of NADH dehydrogenase subunits: NDUFA6: 1.043 ± 0.043 (BHHF) vs 0.311 ± 0.099 (model, *P* < 0.0001); NDUFA10: 1.108 ± 0.059 (BHHF) vs 0.719 ± 0.172 (model, *P* = 0.0007); and NDUFA12: 1.146 ± 0.047 (BHHF) vs 0.414 ± 0.095 (model, *P* < 0.0001). These findings were validated by western blot analysis (Fig. 5L). Given the role of Complex I in NADH oxidation, mitochondrial redox states were assessed. The NAD +/NADH redox ratio increased significantly in the BHHF group compared to the model group (0.919 ± 0.222 vs 0.031 ± 0.022, *P* = 0.0139) (Fig. 5P). This enhanced redox capacity was consistent with the observed improvement in Complex I activity, indicating restored electron flux through the ETC.

### BHHF suppressed lipid accumulation via AMPK-mediated mitochondrial bioenergetic reprogramming

**Fig. 6:**
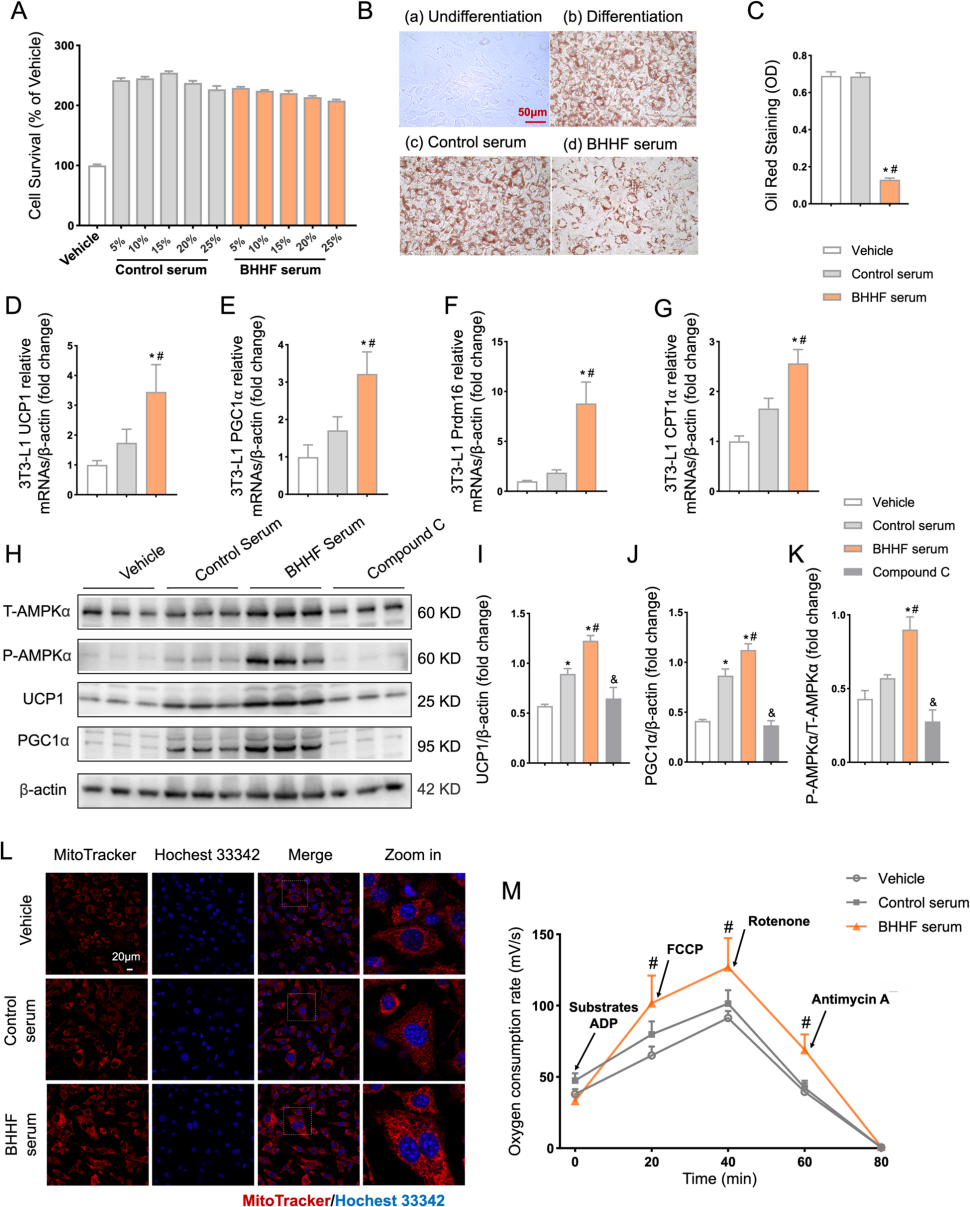
Pharmacological induction of adipocyte browning and mitochondrial remodeling in vitro. **A**: Cytocompatibility assessment of control serum (0–20% v/v) and BHHF-containing serum (0–20% v/v) using the CCK-8 assay after 48-h exposure. **B**: Representative Oil Red O-stained images of 3T3-L1 adipocytes (scale bar: 200 μm) indicating lipid droplet dynamics. **C**: Quantification of intracellular lipid content by isopropanol-eluted Oil Red O absorbance. **D**–**G**: RT-qPCR analysis of thermogenic markers normalized to β-actin: UCP1, PGC-1α, Prdm16, and CPT1a. **H**: Western blot analysis of UCP1, PGC1α, T-AMPKα, and phosphorylated AMPKα (P-AMPKα). **I–K**: Quantification of protein expression ratios: UCP1/β-actin, PGC1α/β-actin, and P-AMPKα/T-AMPKα. **L**: Mitochondrial density visualization using MitoTracker™ Red CMXRos (scale bar: 20 μm). **M**: Oxygen consumption rate (OCR) profiles of 3T3-L1 cells measured by Oroboros O2K. Arrows denote sequential additions of mitochondrial substrates for assessing respiratory states. All data are expressed as mean ± SEM (n = 3 per group). Statistical significance was determined using one-way ANOVA with Tukey’s post hoc test (^*^P < 0.05 vs. vehicle; ^#^*P* < 0.05 vs. control serum; ^&^P < 0.05 vs. BHHF serum)

Cytocompatibility assessment using the CCK-8 assay confirmed that BHHF-containing serum (up to 25% v/v) exhibited no cytotoxicity. A 10% concentration was selected for subsequent experiments (Fig. 6A). As demonstrated by Oil Red O staining, BHHF serum reduced oleic acid-induced lipid droplet accumulation in 3T3-L1 cells (Figs. 6B–C). RT-qPCR revealed significant upregulation of thermogenic markers (normalized to β-actin) in BHHF-treated cells: UCP1: 3.451 ± 2.412 vs 1.742 ± 1.363 (control serum, *P* = 0.0369); PGC1α: 3.218 ± 1.773 vs 1.711 ± 0.956 (control serum, *P* = 0.035); PRDM16: 8.805 ± 5.655 vs 1.864 ± 0.825 (control serum, *P* = 0.0001); and CPT1α: 2.563 ± 0.837 vs 1.660 ± 0.538 (control serum, *P* = 0.0096) (Figs. 6D–G). Pharmacological inhibition of AMPK with compound C (10 μM) attenuated BHHF-induced effects (Figs. 6H–K). MitoTracker™ Red CMXRos staining revealed increased mitochondrial density, indicating mitochondrial biogenesis and enhanced functionality (Fig. 6L). High-resolution respirometry using the Oroboros O2k system quantified mitochondrial bioenergetic remodeling: OXPHOS capacity: 117.4 ± 29.43 (BHHF serum) vs 72.97 ± 24.72 mVO_2_/s (control serum, *P* = 0.0102); electron transfer capacity: 143.9 ± 28.82 (BHHF serum) vs 94.42 ± 25.41 mVO_2_/s (control serum, *P* = 0.0043); and S-pathway (fatty acid oxidation): 76.27 ± 20.84 (BHHF serum) vs 41.80 ± 10.14 mVO_2_/s (control serum, *P* = 0.0034) (Fig. 6M). These findings suggest that the AMPK–mitochondria axis synergistically reprogrammed energy flux toward thermogenesis rather than lipid storage.

### Systematic deconstruction of BHHF revealed functional specificity of herbal components

**Fig. 7:**
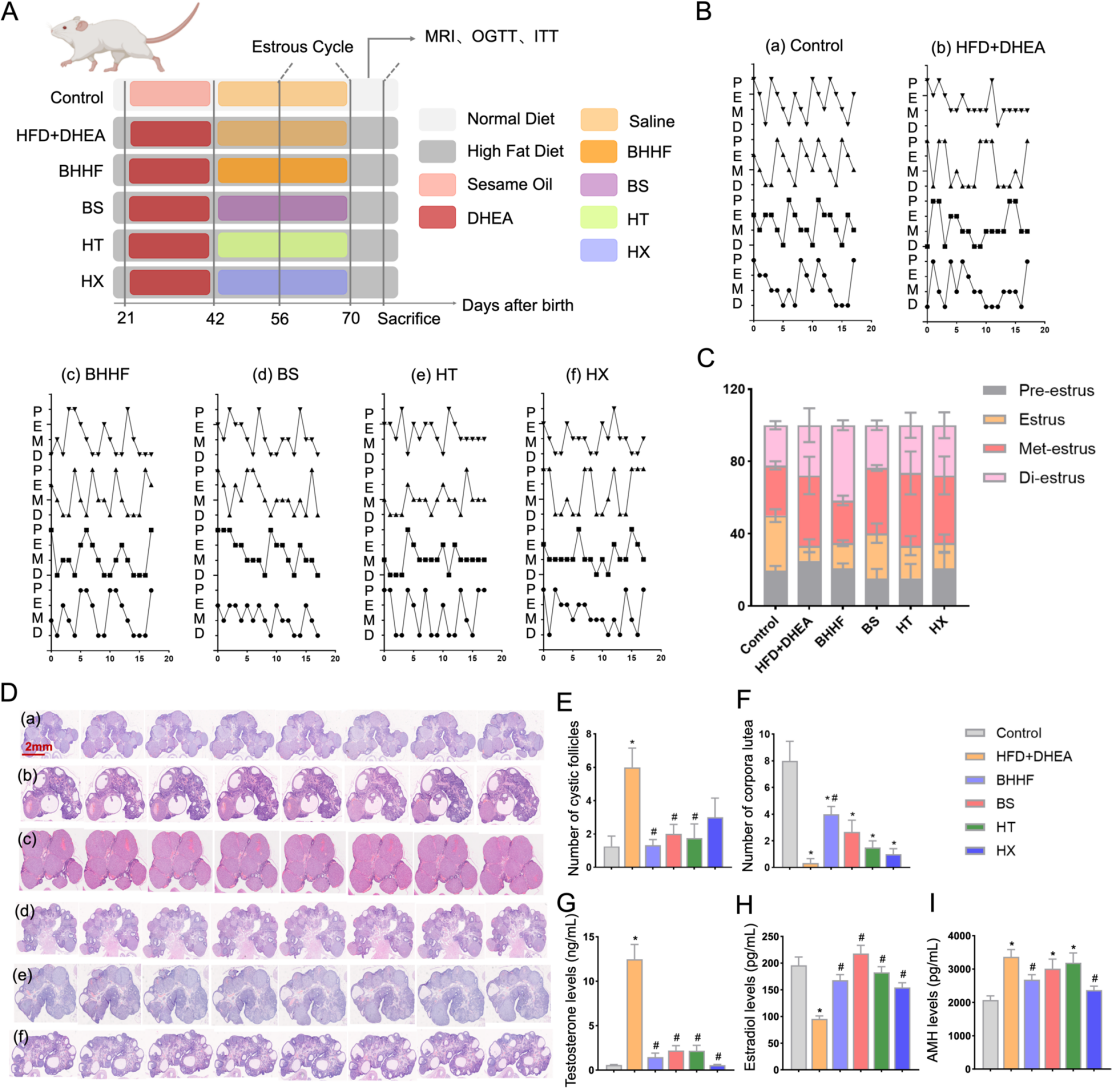
Reproductive cycle characteristics, ovarian histomorphometry, and endocrine profiles. **A**: Schematic illustration of PCOS with obesity rat model establishment and the experimental intervention timeline. **B**: Representative vaginal cytology images across estrous cycle phases: pre-estrus, estrus, met-estrus, and di-estrus.** C**: Quantitative analysis of estrous cycle regularity. **D**: H&E-stained ovarian sections displaying follicular development (scale bar: 2 mm). Groups: (a) Control, (b) HFD + DHEA, (c) BHHF, (d) BS, (e) HT, (f) HX. **E**: Cystic follicle counts per ovarian section. **F**: Corpus luteum counts per ovarian section. **G–I**: Serum hormone levels: testosterone (G), estradiol (H), and anti-Müllerian hormone (AMH) (I).All data are expressed as mean ± SEM (n = 4 per group). Statistical significance was determined using one-way ANOVA with Tukey’s post hoc test. (^*^P < 0.05 vs. control, ^#^*P* < 0.05 vs. HFD + DHEA).

The experimental design for pharmacological deconstruction of BHHF into BuShen (BS), HuaTan (HT), and HX components is outlined in Fig. 7A and Table S2. BS treatment significantly prolonged estrus duration (25.03 ± 10.63% vs. 8.375 ± 7.191% in the model group, *P* = 0.0123), recapitulating BHHF's core efficacy (Figs. 7B–C). H&E staining of ovarian sections demonstrated distinct therapeutic contributions: BHHF, BS, and HT reduced cystic follicles by 77.78% (1.333 ± 0.577 vs. 6.000 ± 2.000, *P* = 0.0018), 66.67% (2.000 ± 1.155 vs. 6.000 ± 2.000, *P* = 0.0034), and 70.83% (1.750 ± 1.708 vs. 6.000 ± 2.000, *P* = 0.0022), respectively (Figs. 7E–F). Serum profiling revealed component-specific endocrine reprogramming: testosterone was reduced by 88.18% (1.474 ± 0.888 in BHHF vs. 12.47 ± 4.072 in the model group, *P* < 0.0001); BS and HT exhibited comparable efficacy (Δ = 82.35–82.49%, *P* = 0.9879), while the HX group demonstrated greater suppression (Δ = 95.41%, *P* < 0.0001) (Fig. 7G). All treatment groups elevated estradiol levels (BHHF: 1.76-fold vs. model, *P* < 0.0001; BS: 2.28-fold, *P* < 0.0001; HT: 1.91-fold, *P* < 0.0001; and HX: 1.61-fold, *P* = 0.0024) (Fig. 7H). Both BHHF and HX uniquely reduced AMH levels (2682 ± 301.8 vs. 3368 ± 525.2, *P* = 0.0446; and 2369 ± 270.9 vs. 3368 ± 525.2, *P* = 0.0030, respectively) (Fig. 7I).

### HT component regulated glucose and lipid metabolic homeostasis in PCOS with obesity

**Fig. 8:**
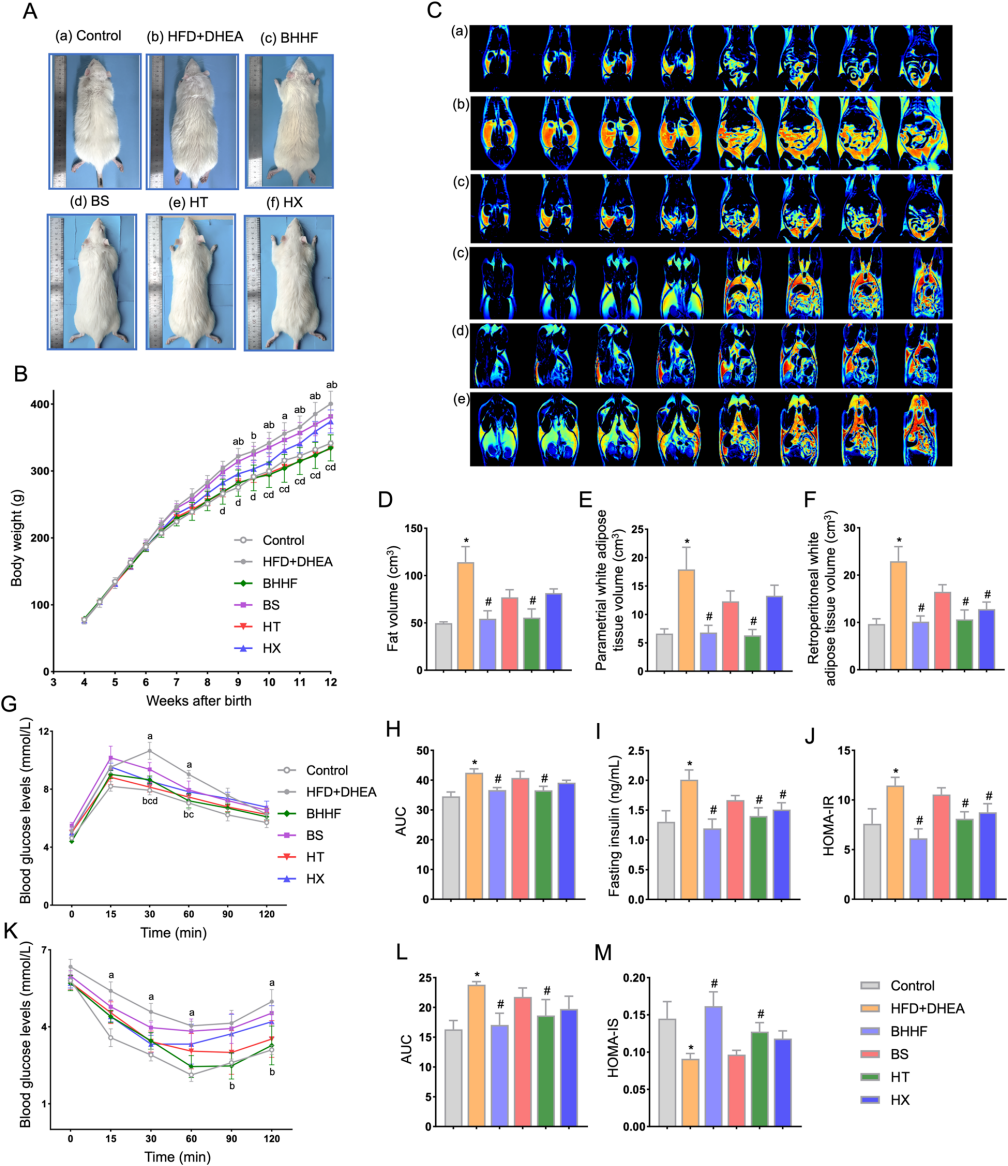
Systemic glucose and lipid metabolic profiles. **A**: Gross morphology of rats. **B**: Longitudinal body weight monitoring. (a *P* < 0.05 Control vs. HFD + DHEA; b *P* < 0.05 Control vs. BS; c *P* < 0.05 HFD + DHEA vs. BHHF; d *P* < 0.05 HFD + DHEA vs. HT) **C**: Representative MRI cross-sectional scans: left four panels show retroperitoneal adipose tissue; right four panels show parametrial adipose tissue. Groups: (a) Control, (b) HFD + DHEA, (c) BHHF, (d) BS, (e) HT, (f) HX. **D–F**: Quantitative MRI adipose analysis—total fat volume (D), parametrial fat volume (E), and retroperitoneal fat volume (F). **G**: Oral glucose tolerance test (OGTT) curves over 0–120 min. (a *P* < 0.05 Control vs. HFD + DHEA; b *P* < 0.05 HFD + DHEA vs. BHHF; c *P* < 0.05 HFD + DHEA vs. HT; d *P* < 0.05 HFD + DHEA vs. HX) **H**: Area under the curve (AUC) of OGTT. **I**: Serum insulin levels after 12-h overnight fasting. **J**: HOMA-IR calculation. **K**: Insulin tolerance test (ITT) curves over 0–120 min. (a *P* < 0.05 Control vs. HFD + DHEA; b *P* < 0.05 HFD + DHEA vs. BHHF) **L**: AUC of ITT. **M**: HOMA-IS calculation. All data are expressed as mean ± SEM (n = 4 per group). Statistical significance was determined by one-way ANOVA with Tukey’s post hoc test. (^*^P < 0.05 vs. control, ^#^*P* < 0.05 vs. HFD + DHEA)

**Fig. 9:**
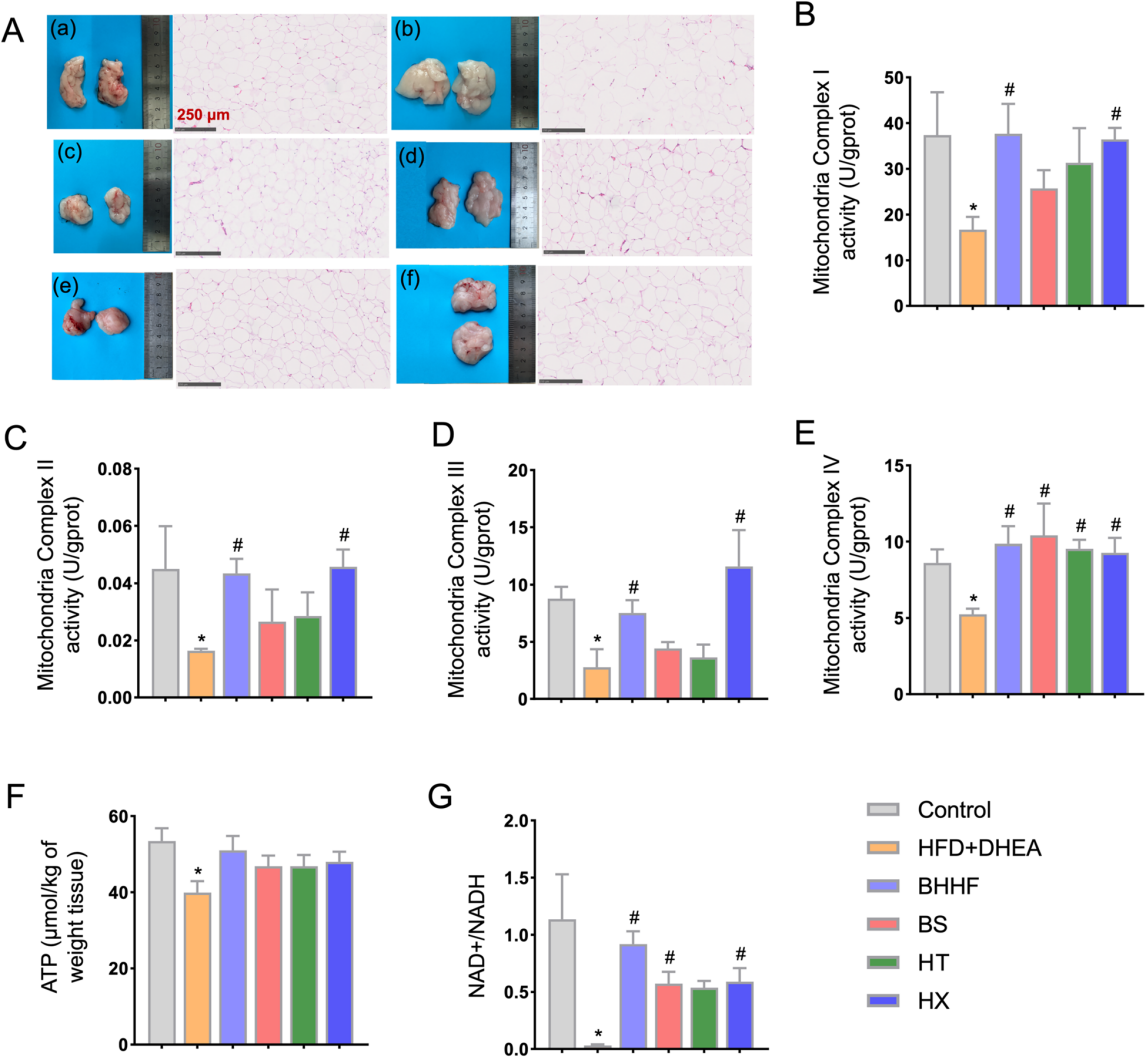
Adipocyte browning and mitochondrial complex activities. **A**: Macroscopic adipose depot morphology (left) and H&E staining (right; scale bars: 250 μm). **B–E**: Mitochondrial electron transport chain (ETC) complex activities in adipose tissue—Complex I (NADH dehydrogenase), Complex II (succinate dehydrogenase), Complex III (ubiquinol-cytochrome c reductase), and Complex IV (cytochrome c oxidase). **F**: Adipose ATP content. **G**: Adipose NAD +/NADH ratio, indicating redox shift. All data are expressed as mean ± SEM (n = 3 per group). Statistical significance was determined by one-way ANOVA with Tukey’s post hoc test. (^*^P < 0.05 vs. control, ^#^*P* < 0.05 vs. HFD + DHEA)

Longitudinal monitoring revealed that HT significantly attenuated weight gain (Δ body weight at week 12: 255.8 ± 4.563 g vs 324.8 ± 25.38 g in the model group, *P* = 0.0141), whereas BS and HX demonstrated limited efficacy (*P* = 0.413 for BS vs model; *P* = 0.278 for HX vs model) (Fig. 8A–B). MRI-based quantification showed that HT treatment led to a 51.37% reduction in total fat volume compared to the model group (55.54 ± 20.43 vs 114.2 ± 32.71 cm^3^, *P* = 0.0001). Parametrial fat volume was reduced to 6.317 ± 2.300 cm^3^ (vs 17.92 ± 7.799 cm^3^, *P* = 0.0004), and retroperitoneal fat volume to 10.64 ± 3.992 cm^3^ (vs 22.93 ± 6.167 cm^3^, *P* = 0.0002) (Fig. 8D–F). Histological examination of parametrial adipose tissue revealed HT-induced morphological remodeling (Fig. 9A). Furthermore, HT recapitulated the metabolic benefits observed with BHHF, as indicated by systemic glycemic improvement: glucose tolerance AUC was reduced by 13.90% compared to the model group (*P* = 0.0184) (Fig. 8G–H), and insulin sensitivity AUC was reduced by 21.70% (*P* = 0.048) (Fig. 8K–L). Notably, HT achieved 66% and 52% of BHHF’s maximal metabolic efficacy in terms of HOMA-IR (8.111 ± 1.576 vs 11.45 ± 2.364 in the model group, *P* = 0.0089) and HOMA-IS (0.1275 ± 0.027 vs 0.091 ± 0.020, *P* = 0.0273), respectively, positioning HT as the primary glucolipid-modulating component in the formula (Fig. 8J and M).

### HX component modulated mitochondrial bioenergetics via enhancing ETC activity and cellular redox homeostasis

As illustrated in Figs. 9B–E, targeted analysis of ETC complexes revealed HX-induced functional restoration. HX significantly enhanced the enzymatic activities of mitochondrial respiratory chain complexes I (NADH dehydrogenase, *P* = 0.048), II (succinate dehydrogenase, *P* = 0.0317), III (ubiquinol–cytochrome c reductase, *P* = 0.0014), and IV (cytochrome c oxidase, *P* = 0.0205) compared to the model group. Concomitant with ETC enhancement, HX shifted the mitochondrial NAD^+^/NADH redox balance, increasing the NAD^+^/NADH ratio from 0.03 ± 0.022 (model) to 0.59 ± 0.263 (*P* = 0.04) (Fig. 9G), indicating improved oxidative phosphorylation efficiency.

## Discussion

Our study systematically demonstrated that BHHF, a multi-component TCM formula grounded in TCM principles, exerted multi-targeted therapeutic effects in ameliorating both reproductive and metabolic dysfunctions associated with obesity-related PCOS. First, we showed that BHHF restored estrous cyclicity, reduced cystic follicles, and significantly improved hyperandrogenemia. The observed attenuation of hyperandrogenemia and PCOM by BHHF is consistent with emerging evidence suggesting that TCM can modulate GPR54/GnRH signaling via hypothalamic–pituitary–ovarian axis regulation [38]. Furthermore, BHHF’s metabolic efficacy was evidenced by attenuation of weight gain and body fat percentage, induction of adipose tissue browning, restoration of mitochondrial redox balance, improvement in glucose homeostasis, and enhancement of insulin sensitivity. These findings align with recent pharmacological studies emphasizing the importance of addressing both metabolic–reproductive crosstalk and tissue-specific pathophysiology in PCOS [36, 39–41]. Second, our study revealed that the BS component restored regular estrous cycles and improved PCOM. This multi-level regulation is consistent with the TCM principle of"kidney-tonifying,"which historically targets reproductive axis dysfunction. The core herb in the BS component, *Epimedium brevicornum* Maxim., contains icariin—a flavonoid derived from *Epimedium*—which has been shown to exert therapeutic effects on autoimmune premature ovarian insufficiency via modulation of the Nrf2/HO-1/Sirt1 pathway [42]. Third, BHHF ameliorated glucolipid metabolic disorders through HT-mediated adipose browning, which involved AMPKα (Thr172) phosphorylation and upregulation of UCP1 and PGC1α. These effects parallel those of berberine in promoting GLUT4 translocation [43, 44] and enhancing insulin sensitivity and are consistent with recent findings that beige adipogenesis can improve PCOS [45–47]. Fourth, our data demonstrated that the HX component promoted activation of mitochondrial Complexes I–IV, induced bioenergetic remodeling, and restored the NAD^+^/NADH ratio—key mechanisms involved in mitochondrial dysfunction in the pathogenesis of PCOS [48, 49]. Moreover, reduced NAD^+^ levels in skeletal muscle observed in hyperandrogenic PCOS models may contribute to metabolic dysregulation [50].

Crucially, AMPKα phosphorylation played a central role in integrating BHHF’s metabolic and reproductive effects. Our data demonstrated that Thr172-phosphorylated AMPKα mediated the BHHF-induced improvements in both metabolic outcomes and reproductive function. This mechanism aligns with emerging evidence that AMPK activation coordinates lipid oxidation and ovarian steroidogenesis [51, 52], while suppressing CYP17A1-mediated androgen overproduction via the SIRT1/NF-κB signaling pathway [53]. Moreover, the dual regulatory role of AMPK in the energy homeostasis–gonadal axis crosstalk has become a research hotspot in PCOS [54–56]. Activation of the AMPK–PGC1α–UCP1 axis induces beige adipogenesis by enhancing mitochondrial cristae density, and recent studies have reported AMPK-driven mitochondrial remodeling in beige adipocytes [57]. Additional studies have demonstrated that brown adipose tissue xenotransplantation in rats improved multiple indices of follicle and oocyte quality, as well as enhanced metabolic function and general health in aging mice [58]. The coordinated activation of AMPK-driven fatty acid oxidation and NAD^+^ salvage pathway supports the role of BHHF as a multi-targeted intervention in metabolic reprogramming—bridging adipocyte plasticity with systemic energy expenditure regulation.

The current first-line management of PCOS relies on long-term lifestyle interventions, including dietary modification, increased physical activity, and weight reduction [59]. For patients with menstrual irregularities, oral contraceptives are used to regulate follicle-stimulating hormone and luteinizing hormone levels, thereby restoring the menstrual cycle [60]. However, these hormonal therapies may adversely affect metabolic homeostasis, including disturbances in glucose and lipid metabolism, while ovulation induction is associated with increased risks of multiple gestation and ovarian hyperstimulation syndrome [61]. In addition, some patients exhibit poor responses to ovulation induction therapy, and some remain infertile despite ovulation. First-line pharmacological agents such as metformin improve insulin resistance but fail to address hyperandrogenism or ovarian dysfunction, while clomiphene and anti-androgens promote ovulation without providing metabolic benefits [62]. Recent efforts to repurpose glucagon-like peptide-1 (GLP-1) receptor agonists have shown promise for weight reduction but demonstrated limited effects on fertility [21]. Although pharmacological interventions alleviate PCOS symptoms, high relapse rates following treatment discontinuation impose substantial socioeconomic burdens. Most current clinical trials on TCM primarily focus on symptom relief rather than mechanistic evaluation. Cangfu Daotan Decoction, one of the most widely used Chinese herbal formulas for PCOS, contains 12 active ingredients and has been shown to reduce androgen levels and promote ovulation [63]. Similarly, Guizhi Fuling Wan, a TCM formulation targeting the PI3K/AKT pathway, was found to reduce granulosa cell autophagy in PCOS rats by restoring the PI3K/AKT/mTOR signaling axis, although it exhibited limited metabolic benefits [33]. However, many TCM investigations emphasize isolated compounds—such as resveratrol, known for AMPK activation, or berberine, recognized for modulating gut microbiota—while overlooking the synergistic potential of multi-component herbal formulations. Our findings established the multi-component nature of BHHF, emphasized the characterization of PCOS as a metabolic-reproductive syndrome, and highlighted the potential superiority of herbal combinations in addressing PCOS heterogeneity. Nevertheless, identifying the core active components of BHHF remains essential. Emerging technologies may enhance mechanistic insights and formulation optimization: (1) spatial transcriptomics to map ovarian–adipose tissue crosstalk during treatment; (2) organoid models of PCOS theca cells to optimize component ratios for specific PCOS phenotypes; and (3) AI-driven network pharmacology to predict synergistic interactions and refine dosage strategies.

## Conclusion

**Fig. 10:**
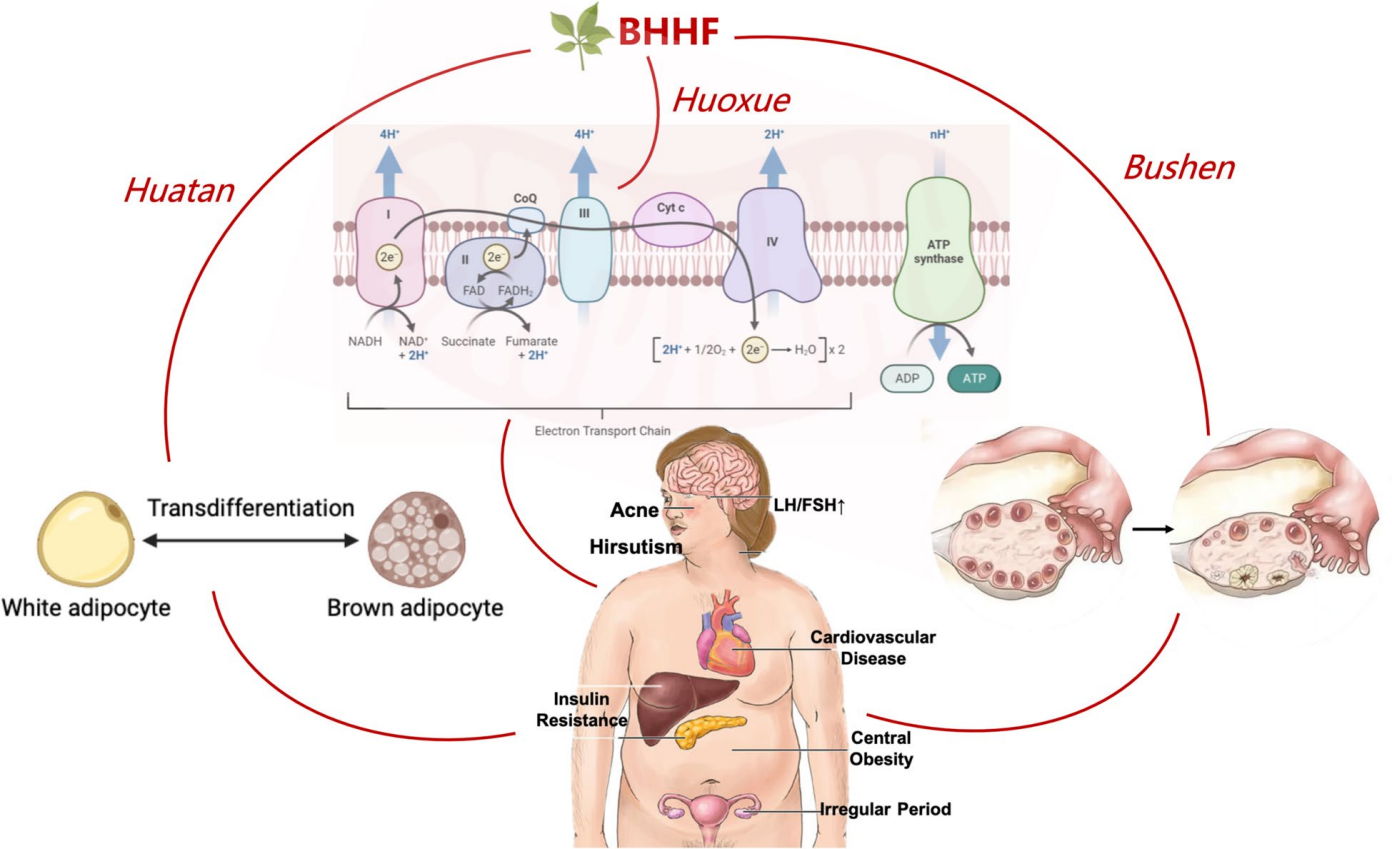
Proposed mechanisms of BHHF and its components in ameliorating obesity-associated PCOS

The differential efficacy of the individual BHHF components—BS for ovarian cyclicity, HT for adipose plasticity, and HX for mitochondrial repair—offers a framework for precision-based TCM (Fig. 10). For example, the predominance of HT in metabolic regulation suggests its potential application in obese PCOS subtypes, whereas HX-mediated restoration of the NAD^+^/NADH ratio may benefit patients with pronounced mitochondrial dysfunction. By integrating TCM principles with modern biotechnological approaches, our findings propose a therapeutic model for PCOS that transitions from symptom management to multi-tissue targeting and outlines actionable molecular pathways for clinical translation.

This tripartite pharmacodynamic architecture elucidates the modern biomedical basis of traditional Chinese medicinal principles through quantifiable, molecular, and pathophysiological targets. This represents a paradigm shift from single-target interventions to multi-targeted strategies, underscoring the necessity for therapies that address multiple pathophysiological pathways concurrently. Future studies incorporating mitochondrial haplotyping and spatial omics technologies are expected to accelerate the progression from empirical herbal formulations to mechanism-driven precision therapies.

## Supplementary Information

Supplementary file 1

## Data Availability

The datasets used and analyzed during the current study are available from the corresponding author upon reasonable request.

## Abbreviations

BHHF: Bushen Huatuan Huoxue Formula
CPT1α: Carnitine palmitoyltransferase 1α
DEPs: Differentially expressed proteins
ELISA: Enzyme-linked immunosorbent assay
FSH: Follicle-stimulating hormone
HOMA-IR: Homeostasis model assessment-insulin resistance
HFD: High fat diet
ITT: Insulin tolerance test
LH: Luteinizing hormone
NCD: Normal chow diet
OCR: Oxygen consumption rates
OGTT: Oral glucose tolerance test
OXPHOS: Oxidative phosphorylation
PGC1α: Peroxisome proliferator-activated receptor-γ coactivator 1-α
PCOM: Polycystic ovarian morphology
PCOS: Polycystic ovary syndrome
TCM: Traditional chinese medicine
UCP1: Uncoupling protein 1
UPLC-MS: UPLC-QE-Orbitrap-MS/MS
RT-qPCR: Real-time quantitative PCR
