# Supplementary material for: Mechanistic insights into the Bushen Huatan Huoxue Formula and its components in ameliorating obesity-associated polycystic ovary syndrome
Source: Chin Med. 2025 Jul 1;20:99. doi: 10.1186/s13020-025-01165-3 (PMC12220228; doi:10.1186/s13020-025-01165-3)
Supplement: Supplementary file 1 [file 13020_2025_1165_MOESM1_ESM.pdf]

Supplementary Tables

Table S1. Composition of the BHHF.

Herb pinyin name	Herb latin name	Use part	Percentage of composition ratio (%)
Yin Yang Huo	*Epimedium brevicornum* Maxim.	Dried aerial	14
Nv Zhen Zi	*Ligustrum lucidum* W.T.Aiton	Fruit	18.7
Tu Si Zi	*Cuscuta chinensis* Lam.	Seed	14
Cang Zhu	*Atractylodes lancea* (Thunb.) DC.	Dried rhizomes	9.3
Huang Lian	*Coptis chinensis* Franch.	Dried rhizomes	5.6
Dan Nan Xing	*Arisaema erubescens*	Fermented rhizome	5.6
Xiang Fu	*Cyperus rotundus* L.	Dried tuberous rhizomes	9.3
Dan Shen	*Salvia miltiorrhiza* Bunge	Dried roots and rhizomes	14
Bai Shao	*Paeonia lactiflora* Pall.	Dried roots	9.3

Table S2. Systematic deconstruction of BHHF

Component	Herb pinyin name	Herb latin name	Percentage of composition ratio (%)
Bushen	Yin Yang Huo	*Epimedium brevicornum* Maxim.	30
	Nv Zhen Zi	*Ligustrum lucidum* W.T.Aiton	40
	Tu Si Zi	*Cuscuta chinensis* Lam.	30
Huatan	Cang Zhu	*Atractylodes lancea* (Thunb.) DC.	45.4
	Huang Lian	*Coptis chinensis* Franch.	27.3
	Dan Nan Xing	*Arisaema erubescens*	27.3
Huoxue	Xiang Fu	*Cyperus rotundus* L.	28.6
	Dan Shen	*Salvia miltiorrhiza* Bunge	42.8
	Bai Shao	*Paeonia lactiflora* Pall.	28.6

Table S3. Compounds in BHHF solution detected by UPLC-MS in positive and negative mode.

Detection mode	Area	Retention time	Adduct	m/z	Library hit
Positive	8269000	9.68	[M+H]+	355.1024	Neochlorogenic acid
Positive	468000	12.22	[M+H]+	611.1607	Rutin
Positive	412800	13.31	[M+H]+	449.1078	Astragalin
Positive	412800	13.31	[M+H]+	449.1078	Luteoloside
Positive	9664000	12.42	[M+H]+	465.1028	Hyperin
Positive	20110000	0.88	[M+H]+	175.119	(+)-Arginine
Positive	1047000	0.93	[M+H]+	148.0604	Glutamic acid
Positive	10080000	16.39	[M+H]+	677.244	Icarrin
Positive	21880	1.37	[M+H]+	279.1016	Linolenic acid
Positive	834600	1.05	[M+H]+	138.055	Trigonelline
Positive	169800000	15.45	[M+H]+	336.123	Berberine
Positive	1149000	3.23	[M+H]+	132.1019	Isoleucine
Positive	9664000	12.42	[M+H]+	465.1028	Isoquercitrin
Positive	157800	13.55	[M+H]+	433.1129	Genistin
Positive	157800	13.55	[M+H]+	433.1129	Sophoricoside
Positive	157800	13.55	[M+H]+	433.1129	Apigenin-7-glucoside
Positive	573500	10.76	[M+H]+	377.1442	Vitamin B2
Positive	642100	12.59	[M+H]+	642.2392	IsoActeoside
Positive	12530000	1.06	[M+H]+	116.0706	Proline
Positive	139100	11.65	[M+H]+	760.3022	Eleutheroside E
Positive	31850000	8.88	[M+H]+	318.1547	Salidroside
Positive	123400	15.27	[M+H]+	314.1387	N-trans-ferulotyramine
Positive	642100	12.59	[M+H]+	642.2392	Acteoside
Positive	642100	12.59	[M+H]+	642.2392	Forsythoside A
Positive	73060	12.96	[M+H]+	595.1657	Aempferol-3-O-rutinoside
Positive	190200	11.27	[M+H]+	700.2811	Pinoresinol Diglucoside

Positive	44720	13.14	[M+H]+	625.1763	Forsythoside I
Positive	1178000	1.17	[M+H]+	144.1019	Stachydrine
Positive	2283000	1.65	[M+H]+	130.0863	Pipecolinic acid
Positive	12060000	22.08	[M+H]+	237.1849	Curdione
Positive	5483000	12.42	[M+H]+	303.0499	Quercetin
Positive	412800	13.31	[M+H]+	449.1078	Cyanidin-3-O-glucoside
Positive	8886	26.3	[M+H]+	295.2632	Tanshinone IIA
Positive	1919000	18.46	[M+H]+	295.2268	9-oxo-10(E),12(E)-octadecadienoic acid
Positive	12060000	22.08	[M+H]+	237.1849	Curcumol
Positive	1919000	18.46	[M+H]+	295.2268	13-oxo-9(E),11(E)-octadecadienoic acid
Positive	1149000	3.23	[M+H]+	132.1019	Leucine
Positive	452000	13.37	[M+H]+	317.0656	Isorhamnetin
Positive	1919000	18.46	[M+H]+	295.2268	9-oxo-octadeca-5(E),7(E)-dienoic acid
Positive	163200	12.55	[M+H]+	359.1489	Pinoresinol
Positive	1919000	18.46	[M+H]+	295.2268	13(R)-hydroxy-octadeca (9Z,11E,15Z)-trienoic acid
Positive	10670000	11.32	[M+H]+	369.118	Curcumin
Positive	452	10.95	[M+H]+	342.0804	Magnoflorine
Positive	163900	13.4	[M+H]+	579.1708	Rhoifolin
Positive	845000	18.47	[M+H]+	195.138	Sedanolide
Positive	82070	12.79	[M+H]+	223.0601	Isofraxidin
Positive	90670	25.8	[M+H]+	297.2424	Cryptotanshinone
Positive	5483000	12.42	[M+H]+	303.0499	Morin hydrate
Positive	4722000	26.73	[M+H]+	391.2843	Loganin
Positive	82070	12.79	[M+H]+	223.0601	8-hydroxy-6,7-dimethoxycoumarin
Positive	92340	14.58	[M+H]+	207.0652	Scoparone

Positive	1580	7.98	[M+H]+	151.0601	(-)-Carvone
Positive	92340	14.58	[M+H]+	207.0652	Dimethoxycoumarin
Positive	288700	10.33	[M+H]+	147.0441	Coumarin
Positive	470000	13.32	[M+H]+	153.1274	Pulegone
Positive	10060	26.13	[M+H]+	239.2369	Muscone
Positive	384500	13.31	[M+H]+	287.055	Kaempferol
Positive	12490	14.72	[M+H]+	225.1121	Senkyunolide I
Positive	46440	11.97	[M+H]+	149.0961	Anise oil
Positive	31850000	8.88	[M+H]+	318.1547	Salidroide
Positive	188600	2.11	[M+H]+	177.0394	Vitamin C
Positive	33390	10.71	[M+H]+	227.0914	Genipin
Positive	35830	26.19	[M+H]+	383.1965	Campesterol
Positive	72280	2.02	[M+H]+	137.0597	Bomyl acetate
Positive	546600	8.4	[M+H]+	146.06	Indole-3-carboxaldefyde
Positive	416600	15.28	[M+H]+	271.0965	Aloe-Emodine
Positive	78470	22.79	[M+H]+	376.2482	Tuberostemonine
Positive	179800	12.45	[M+H]+	177.0546	Methoxycoumarin
Positive	46800	13.36	[M+H]+	419.17	Syringaresinol
Positive	38530	8.69	[M+H]+	197.0808	Cantharidin
Positive	303900	26.01	[M+H]+	285.1697	Lupinalbin A
Positive	92340	14.58	[M+H]+	207.0652	Limettin
Positive	46800	13.36	[M+H]+	419.17	Episyringaresinol
Positive	125600	7.47	[M+H]+	170.0812	Vitamin B6
Positive	609	10.08	[M+H]+	257.0444	Bakuchiol
Negative	47840000	14.57	[M-H]-	717.1461	Salvianolic acid B
Negative	123000	11.64	[M-H]-	787.2666	Eleutheroside E
Negative	788800	1.34	[M-H]-	115.0037	Maleic acid
Negative	788800	1.34	[M-H]-	115.0037	Fumaric acid
Negative	83270	20.51	[M-H]-	825.4642	Saikosaponin D
Negative	1643000	7.19	[M-H]-	153.0193	Protocatechuic acid

Negative	41080000	2.13	[M-H]-	191.0197	Citric acid
Negative	12360	11.11	[M-H]-	681.2917	Pinoresinol Diglucoside
Negative	1313000	13.06	[M-H]-	623.1981	Verbascoside
Negative	1313000	13.06	[M-H]-	623.1981	IsoActeoside
Negative	182900	0.88	[M-H]-	132.0302	Aspartic acid
Negative	187200	13.55	[M-H]-	431.0984	L-Tryptophan
Negative	2123000	8.41	[M-H]-	203.0826	Sophoricoside
Negative	187200	13.55	[M-H]-	431.0984	Glutamic acid
Negative	669400	0.91	[M-H]-	146.0459	Salvianolic acid D
Negative	447600	7.41	[M-H]-	359.0772	Rosmarinic acid
Negative	447600	7.41	[M-H]-	359.0772	Luteoloside
Negative	1002000	13.31	[M-H]-	447.0933	Luteolin-7-O-β-D-glucoside
Negative	127800	8.41	[M-H]-	197.0455	Danshensu
Negative	127800	8.41	[M-H]-	197.0455	Lonicerin
Negative	32850	10.42	[M-H]-	153.0557	Quinic acid
Negative	37320	1.39	[M-H]-	191.0561	Ellagic Acid
Negative	39730	12.34	[M-H]-	300.999	Ligustroflavone
Negative	97860	3.17	[M-H]-	723.2142	Wogonin
Negative	1286	26.02	[M-H]-	283.1551	Uridine
Negative	1860	1.91	[M-H]-	243.1027	Phellodendrine
Negative	2110	10.8	[M-H]-	340.0658	Daidzin
Negative	15280	9.54	[M-H]-	491.1923	Tubuloside A
Negative	12280000	11.31	[M-H]-	367.1187	Emodin-8-glucoside
Negative	187200	13.55	[M-H]-	431.0984	Paederoside
Negative	14360	11.78	[M-H]-	491.1559	Maltopentaose
Negative	548900	1.54	[M-H]-	827.2674	Biochanin A [Smart Confirmation]
Negative	3997	15.97	[M-H]-	283.0612	Isorhamnetin-3-O-neohespeidoside
Negative	49870	13.14	[M-H]-	623.1618	Acacetin

Table S4. Compounds in BHHF serum detected by UPLC-MS in positive and negative mode.

Detection mode	Area	Retention time	Adduct	m/z	Library hit
Positive	57150	0.88	[M+H]+	120.0655	D-Threonine
Positive	173000	1.23	[M+H]+	244.0928	Cytidine
Positive	1694000	1.23	[M+H]+	123.0553	Nicotinamide
Positive	54140	25.86	[M+H]+	279.2319	Linolenic acid
Positive	35300	0.85	[M+H]+	156.0768	Histidine
Positive	33430	1.02	[M+H]+	138.055	Trigonelline
Positive	996800	1.02	[M+H]+	116.0706	Proline
Positive	1312000	1.91	[M+H]+	132.1019	Leucine
Positive	158600	1.1	[M+H]+	144.1019	Stachydrine
Positive	1312000	1.91	[M+H]+	132.1019	Isoleucine
Positive	1511	4.87	[M+H]+	137.0458	Bornyl acetate
Positive	671	0.88	[M+H]+	130.0321	Pipecolinic acid
Positive	5633	23.88	[M+H]+	167.0703	Ethylvanillin
Positive	120600	1.27	[M+H]+	147.0441	Coumarin
Positive	165	22.95	[M+H]+	376.2482	Tuberostemonine
Positive	1187	0.89	[M+H]+	383.1521	Yuheinoside
Positive	73800	20.16	[M+H]+	391.2843	Loganin
Positive	798100	1.26	[M+H]+	165.0546	Coumaric acid
Positive	161000	0.89	[M+H]+	127.039	Hydroxymethylfurfural
Positive	7822	0.85	[M+H]+	170.0812	Lupinine
Positive	45440	25.86	[M+H]+	297.2424	α-dimorphecolic acid
Positive	79090	0.93	[M+H]+	176.103	Citrulline
Positive	885	0.86	[M+H]+	130.0321	Pipecolinic acid
Positive	809	26.48	[M+H]+	385.3465	Resibufogenin
Positive	670600	1.24	[M+H]+	123.0441	4-hydroxy-benzaldehyde
Positive	3650	26.64	[M+H]+	237.1849	Curcumol

Positive	3650	26.64	[M+H]+	237.1849	Curdione
Positive	10700	26.18	[M+H]+	443.3884	Betulin
Positive	13280	25.86	[M+H]+	432.3472	Verticine
Positive	13280	25.86	[M+H]+	432.3472	Peimine
Negative	84230	1.2	[M-H]-	191.0197	Citric acid
Negative	48370	0.87	[M-H]-	124.0074	Taurine
Negative	35520	0.91	[M-H]-	179.0561	D-(+)-Glucose
Negative	1768	1.24	[M-H]-	133.0142	L-Malic acid
Negative	317800	1.2	[M-H]-	175.0248	Vitamin C
Negative	35520	0.91	[M-H]-	179.0561	D-(+)-Mannose
Negative	388	11.76	[M-H]-	429.2283	Resibufogenin
Negative	722	19	[M-H]-	315.233	Isorhamnetin
Negative	32980	0.96	[M-H]-	307.0976	Bisdemethoxycurcumin
Negative	564	11.61	[M-H]-	399.1813	Regaloside A
Negative	308	1.24	[M-H]-	325.0929	Bilobalide
Negative	959	15.3	[M-H]-	283.1551	Biochanin A
Negative	350	11.58	[M-H]-	269.0455	Apigenin
Negative	457	12.1	[M-H]-	483.2388	Pachymic acid B
Negative	2377	26.05	[M-H]-	221.0819	Dehydrovomifoliol

Table S5. Sequences of primers used for RT-qPCR.

Gene name	Gene Forward Primer (5'-3')	Gene Reverse Primer (5'-3')
Cpt1α	CTCCGCCTGAGCCATGAAG	CACCAGTGATGATGCCATTCT
Pgc1α	TATGGAGTGACATAGAGTGTGCT	CCACTTCAATCCACCCAGAAAG
Prdm16	TGCTGACGGATACAGAGGTGT	CCACGCAGAACTTCTCGCTAC
UCP1	AGCCATCTGCATGGGATCAAA	GGGTCGTCCCTTTCCAAAGTG
β-actin	GTGACGTTGACATCCGTAAAGA	GCCGGACTCATCGTACTCC

Supplementary Figures

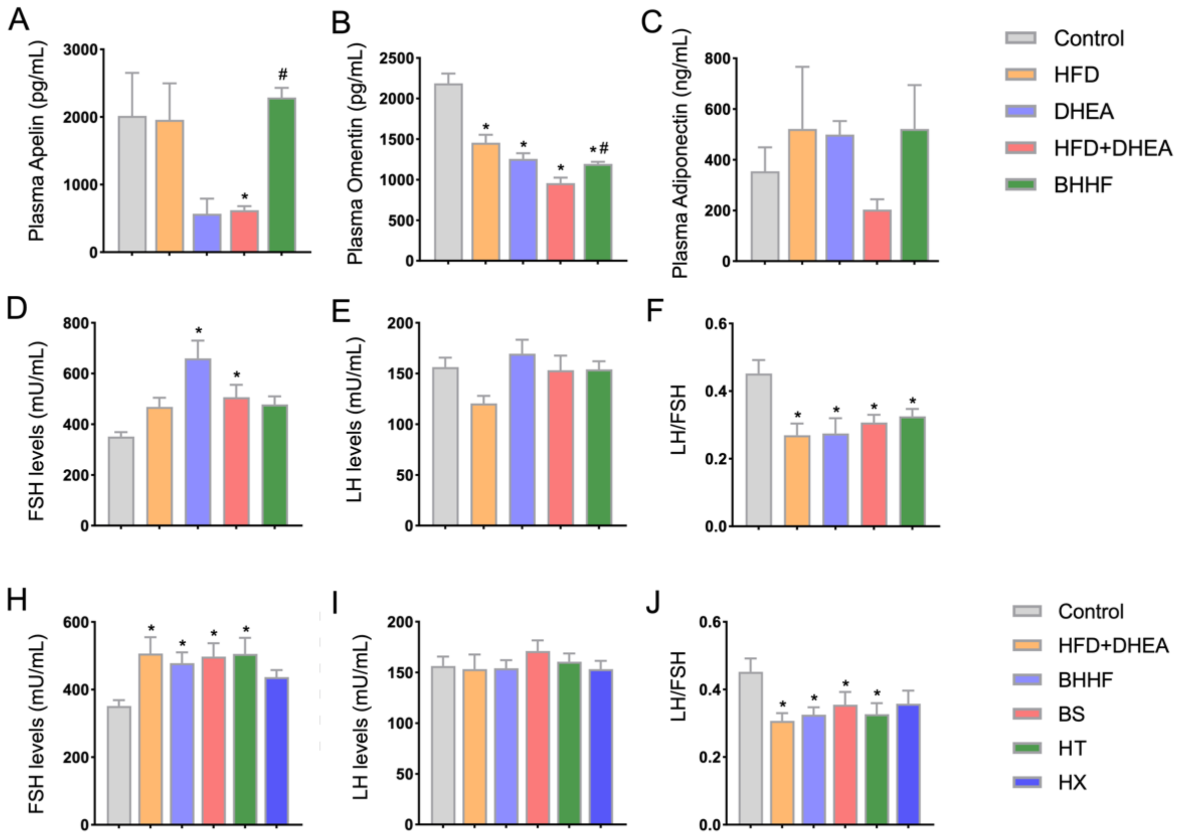

Fig. S1. Systemic endocrine-metabolic profiling of PCOS with obesity. **A:** Circulating apelin contents. **B:** Circulating omentin contents. **C:** Circulating adiponectin contents. **D-I:** Serum hormone levels measured by ELISA: **(D, H)** FSH levels. **(E, I)** LH levels. **(F, J)** LH/FSH ratio. All data expressed as mean ± SEM. n=4 for each group. Statistical significance determined by one-way ANOVA with Tukey's post-hoc test ($^{*}P$<0.05 vs. control, $^{\#}P$<0.05 vs. HFD+DHEA).

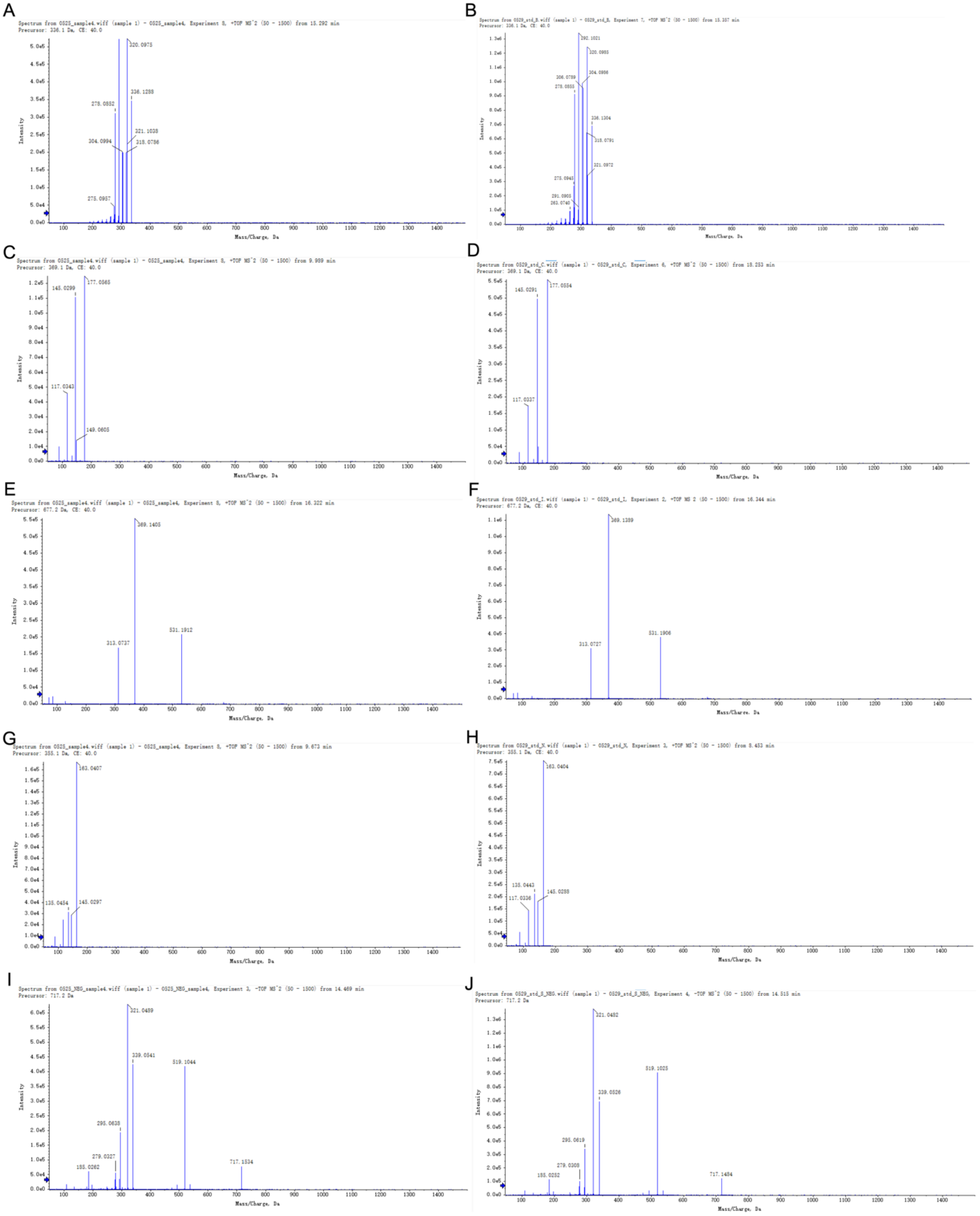

Fig. S2. MS/MS spectra of BHHF solution and representative five standards. Collision-induced dissociation (CID) MS² spectra of deprotonated ($[M\text{-}H]^-$) or protonated ($[M+H]^+$) molecular ions

for BHHF solution and representative five standards. **A:** BHHF solution: MS^2 of *m/z* 336.1 ([M+H]+). **B:** Reference standard of Berberine: MS^2 of *m/z* 336.1 ([M+H]+). **C:** BHHF solution: MS^2 of *m/z* 369.1 ([M+H]+). **D:** Reference standard of Curcumin: MS^2 of *m/z* 369.1 ($[M+H]^-$). **E:** BHHF solution: MS^2 of *m/z* 677.2 ([M+H]+). **F:** Reference Standard of Icariin: MS^2 of *m/z* 677.2 ([M+H]+). **G:** BHHF solution: MS^2 of *m/z* 355.1 ([M+H]+). **H:** Reference Standard of Neochlorogenic acid: MS^2 of *m/z* 355.1 ([M+H]+). **I:** BHHF solution: MS^2 of *m/z* 717.2 ($[M-H]^-$). **J:** Reference Standard of Salvianolic acid B: MS^2 of *m/z* 717.2 ($[M-H]^-$).
